# Diagnostic algorithm for the detection of carbapenemases and extended-spectrum β-lactamases in carbapenem-resistant *Pseudomonas aeruginosa*

**DOI:** 10.1128/spectrum.03196-24

**Published:** 2025-04-16

**Authors:** Stefano Mancini, Laia Garcia-Verellen, Helena M. B. Seth-Smith, Peter M. Keller, Natalia Kolesnik-Goldmann, Muhammad Ali Syed, Irfan Ullah, Vladimira Hinic, Tim Roloff, Adrian Egli, Oliver Nolte

**Affiliations:** 1 Institute of Medical Microbiology, University of Zurich 27217, Zürich, Switzerland; 2 University Hospital Basel, University of Basel 27209, Basel, Switzerland; 3 Department of Microbiology, The University of Haripur 384987, Haripur, Khyber Pakhtunkhwa, Pakistan; University of Kentucky, Lexington, Kentucky, USA

**Keywords:** CRPA, carbapenemase production, carbapenemase detection, ESBL detection

## Abstract

*Pseudomonas aeruginosa* can acquire carbapenem resistance through various mechanisms, including genomic mutations leading to the overexpression of efflux pumps, intrinsic AmpC-β-lactamase, and/or reduced permeability, and/or through the acquisition of plasmid-mediated carbapenemases and/or extended-spectrum-β-lactamases (ESBLs). Unfortunately, carbapenemase/ESBL-producing-carbapenem-resistant-*P. aeruginosa* (CP/ESBL-CRPA) cannot be differentiated from non-CP/ESBL-CRPA based solely on susceptibility testing results of conventional β-lactam (BL)-antibiotics. Knowing that these two groups display different activity profiles toward novel BL/β-lactamase-inhibitor (BLI) combinations, we developed and verified a cost-effective and easy-to-use diagnostic algorithm for screening and differentiation of carbapenemase and ESBL production in CRPA. We determined disc diffusion inhibition zones and gradient strip minimal inhibitory concentration values of 136 whole-genome-sequenced CRPA (70 metallo-β-lactamase-[MBL-], 1 GES-5-, 1 KPC-2-, 12 ESBL-, and 53 AmpC-hyper-producing isolates). We used the following BL-BLI combinations: ceftolozane-tazobactam (C-T), ceftazidime-avibactam, imipenem-relebactam (I-R), meropenem-vaborbactam, cefepime-enmetazobactam (C-E), and aztreonam-avibactam. We also included a lateral flow immunoassay (Carba-5, NG-Biotech) for confirmation of MBL production and double disc synergy testing (DDST) to improve ESBL detection. C-T was the most effective screening antibiotic for distinguishing MBL and ESBL producers from AmpC-hyperproducing CRPA, achieving a sensitivity of 100% for both MBL and ESBL producers. I-R reliably confirmed MBL production in C-T positive screened CRPAs, with a sensitivity of 92.8% and specificity of 100%. Incorporating Carba-5 into the phenotypic algorithm improved sensitivity for confirming MBL production to 100%. For the remaining C-T positive but I-R negative isolates, C-E showed 75% sensitivity and 78.6% specificity in detecting ESBL production. The DDST further confirmed ESBL production in six out of nine ESBL producers (66.6%). In conclusion, we established a simple and cost-effective diagnostic algorithm, enabling screening and confirmation of carbapenemase and ESBL production in CRPA.

**IMPORTANCE:**

Carbapenem-resistant *Pseudomonas aeruginosa* (CRPA) is a major global health threat, and rapid identification of its resistance mechanisms is crucial for effective treatment and infection control. Differentiating between carbapenemase-producing (CP), extended-spectrum β-lactamase-producing (ESBL), and AmpC-hyperproducing CRPA is challenging, as conventional susceptibility testing cannot reliably distinguish these resistance mechanisms. Our study presents a simple, cost-effective, and easy-to-implement phenotypic diagnostic algorithm that enables accurate screening and confirmation of CP and ESBL production in CRPA. This method is particularly valuable for laboratories lacking access to molecular diagnostics, as it provides a practical alternative for routine testing. By facilitating the early detection of resistant *P. aeruginosa* strains, this approach has the potential to improve patient outcomes, optimize antimicrobial therapy, and enhance global surveillance efforts against multidrug-resistant pathogens.

## INTRODUCTION

*Pseudomonas aeruginosa* is a major pathogen causing healthcare-associated infections, especially in immunocompromised and critically ill patients. It is estimated that *P. aeruginosa* infections contribute to over 300,000 deaths annually, and the World Health Organization has classified carbapenem-resistant *P. aeruginosa* (CRPA) among the top critical pathogens posing the greatest threat to human health (1). Carbapenems are considered first-line antibiotics for treating severe *P. aeruginosa* infections (2). *P. aeruginosa* can develop carbapenem resistance through acquisition of plasmid-based carbapenemases or through genomic mutations resulting in the overproduction or increased activity of intrinsic β-lactamases, reduced production or loss of function of the porin OprD, overproduction of efflux pumps (i.e., MexAB-OprN), or a combination of these mechanisms (3). The most common carbapenemase types found in *P. aeruginosa* are Ambler class B metallo-β-lactamases (MBL) including the Verona Imipenemase (VIM), Imipenemase (IMP), German Imipenemase (GIM), and New Delhi metallo β-lactamase (NDM) (4). Other less common carbapenemases are Ambler class A carbapenemases including the *Klebsiella pneumoniae* carbapenemase (KPC) and Guyana extended-spectrum (GES)-type enzymes (5). Carbapenem resistance can also result from the production of class A extended-spectrum-β-lactamases (ESBLs), such as Vietnamese extended-spectrum β-lactamases (VEB), GES and *Pseudomonas* extended-resistance (PER), combined with genomic mutations altering antibiotic permeability via porins (6). The detection of carbapenemase and/or ESBL production is crucial for epidemiological and infection control purposes, as these isolates tend to disseminate between patients more readily than carbapenem-resistant, but non-carbapenemase/ESBL-producing *P. aeruginosa* (non-CP/ESBL-CRPA), thereby necessitating the implementation of stricter infection control measures (7). Furthermore, carbapenemase production, particularly of MBLs, has therapeutic implications. These enzymes can impair the efficacy of newly introduced β-lactam/β-lactamase inhibitor (BL-BLI) combinations such as ceftolozane-tazobactam, ceftazidime-avibactam, meropenem-vaborbactam, and imipenem-relebactam, which otherwise demonstrate good activity against non-CP/ESBL-CRPA. Studies show a higher mortality rate in patients with CP/ESBL-CRPA infections compared with non-CP/ESBL-CRPA infections, underscoring the importance of prompt detection of carbapenemase/ESBL production to reduce therapeutic failures (8).

Differentiation between CP/ESBL-CRPA and non-CP/ESBL-CRPA is not feasible during screening, based on susceptibility data of classic, first-line β-lactam antibiotics. Additionally, the detection of carbapenemase production in *P. aeruginosa* is more challenging compared to *Enterobacterales*, for which these tests have been primarily developed and validated (9).

In diagnostic laboratories, molecular methods are considered the gold standard for the confirmation of the presence of antibiotic resistance genes. However, they are expensive and require specific equipment and trained personnel (10, 11). In this context, non-molecular methods or phenotypic tests offer a more favorable cost-benefit ratio. Currently used phenotypic tests to detect carbapenemase production in *P. aeruginosa* include (i) growth-based methods, such as the modified carbapenem inactivation method, which assesses carbapenem-resistance based on growth in the presence of a carbapenem (12, 13); (ii) carbapenem-hydrolysis based colorimetric tests, which detect production of carbapenem degradation products (e.g., Carba NP or Blue Carba) (14); (iii) inhibitor-based tests, such as the combined disc test, which rely on the increase in disc zone diameter in the presence of AmpC and/or carbapenemase-inhibitors (15); (iv) double disc synergy test (DDST), which detects synergy between discs containing imipenem and MBL-inhibitors (16); and (v) lateral flow immunoassay, which can detect carbapenemase enzymes using specific monoclonal antibodies (11). For ESBL detection, phenotypic assays developed for Enterobacterales, such as the DDST, which detects synergy between discs containing third-generation cephalosporins (such as ceftazidime and cefepime) and ESBL-inhibitors, can be used with modifications, such as reducing disc distance or adding higher concentrations of β-lactamase inhibitors, to enhance sensitivity. While these phenotypic and colorimetric methods generally perform well in detecting MBLs, they show reduced accuracy in identifying class A β-lactamases, including carbapenemases and ESBL. This is primarily due to intrinsic AmpC activity, which can interfere with their detection by masking synergistic phenomena typically observed with ESBL producers (17, 18). Additionally, *P. aeruginosa* isolates typically produce different class A ESBLs compared to Enterobacterales, such as GES and VEB types, which may display distinct profiles compared to Enterobacterales (18).

Since differentiation between CP/ESBL-CRPA and non-CP/ESBL-CRPA cannot be achieved based on the susceptibility data of classic β-lactams, indiscriminate testing for carbapenemase or ESBL production in all CRPA cases would result in numerous unnecessary analyses and significantly increased costs (19). Reflecting this complexity, EUCAST has not yet released guidelines on the screening, confirmation, and/or differentiation of carbapenemase/ESBL production in *P. aeruginosa,* as done for Enterobacterales (20). Due to this reason, most diagnostic laboratories do not perform tests for detecting carbapenemases in CRPA. Consequently, the current rates of CP/ESBL-PA remain largely unknown and likely underestimated, despite their critical implications for infection control and therapeutic decision-making.

The aim of the current project was to develop and validate a simple phenotypic algorithm for screening and confirming carbapenemase/ESBL production in CRPA isolates. This method should be suitable for implementation in small- to medium-sized laboratories, as well as in low-income countries.

## MATERIALS AND METHODS

### Strain collection

A total of 136 non-duplicate *P. aeruginosa* strains from various origins were included in this study (Table S1). The collection encompasses 71 carbapenemase-producers (37× VIM, 17× IMP, 8× NDM, 1× KPC-2, 1×AIM-1, and 1× SPM-1, and 1× GES-5, 1× GES-5/NDM, 1× VIM/NDM, 2× IMP/NDM, 1× IMP-26/OXA-181), 12 class A ESBL-only-producers (× GES, 4×VEB, and 3× PER-1), and 53 carbapenem-resistant *P. aeruginosa* clinical strains with no plasmid-borne carbapenemase/ESBL genes. All isolates exhibited resistance toward ceftazidime, cefepime, and/or piperacillin-tazobactam and were resistant to imipenem and/or non-susceptible to the standard dosing regimen (S) to meropenem (Fig. S1). Ninety-three clinical strains were obtained from unique patients and isolated in the routine diagnostic of the Institute of Medical Microbiology, University of Zurich between January 2011 and March 2024. Twenty-two isolates were derived from unique patients in the routine diagnostic of the Division of Clinical Bacteriology and Mycology of the University Hospital of Basel (University of Basel) between January 2013 and March 2024. Ten clinical strains were isolated at the Khyber Teaching Hospital, Peshawar (Pakistan), and 11 were obtained from the panel of diverse *P. aeruginosa* clinical isolates made publicly available by the Walter Reed Army Institute of Research (21).

### Disc diffusion (DD)

The Kirby-Bauer DD method was performed on agar plates containing cation-adjusted Mueller-Hinton (CAMH) following the EUCAST guidelines (22). All isolates were tested for susceptibility toward β-lactams (piperacillin-tazobactam, ceftazidime, cefepime, meropenem, imipenem, aztreonam, ceftazidime-avibactam 10 + 4 µg [EUCAST], ceftazidime-avibactam 30 + 20 µg [CLSI], ceftolozane-tazobactam, imipenem-relebactam, and meropenem-vaborbactam), aminoglycosides (amikacin and tobramycin), and quinolones (ciprofloxacin and levofloxacin, see Table S2). The inhibition inhibition zone diameters were measured with the Sirscan system (i2a) and double-checked by visual inspection (23). All antibiotic discs were from Oxoid Limited (Basingstoke, United Kingdom).

### Detection of ESBL by double DDST

ESBL production was detected using the DDST, as described by Jiang et al. 17. CAMH agar was inoculated with a standardized inoculum (equivalent to a 0.5 McFarland standard) using a sterile cotton swab. Discs containing BL-BLI combinations (imipenem-relebactam, cefepime-enmetazobactam, and amoxicillin-clavulanic acid) were placed 12, 15, and 20 mm apart from discs containing ceftazidime and cefepime. The enhancement in the growth inhibition zones was observed after 18 h of incubation (Table S3).

### Minimal inhibitory concentration (MIC) test strip

The MIC test strip method was performed on regular CAMH-agar plates from bioMérieux, Marcy L’Etoile, France. MICs of ceftazidime-avibactam, ceftolozane-tazobactam, imipenem-relebactam, meropenem-vaborbactam, and aztreonam-avibactam were determined for all the isolates (Table S4). MIC values were rounded up and adjusted to a binary log scale (i.e., 0.002, (…), 128, and 256). All antibiotic gradient strips were purchased from Liofilchem (Roseto degli Abruzzi, Italy).

### Lateral flow immunoassay (LFIA)

The LFIA Carba-5 (NG-Biotech, France) was performed according to the manufacturer’s instructions (Table S1) (24).

### Data analysis

Sensitivity was determined by calculating the ratio of CP/ESBL-CRPA detected using the established screening cut-off and confirmation method to the total number of CP/ESBL-CRPA detected by whole-genome sequencing (WGS). Specificity was calculated as the ratio of non-CP/ESBL-CRPA detected phenotypically to the total number of non-CP/ESBL-CRPA detected by WGS.

### WGS

Bacterial genomic DNA was extracted using the DNeasy Ultraclean Microbial kit (Qiagen, Hilden, Germany) according to the manufacturer’s instructions. Library preparation was performed with the QIAGEN QIASeq FX kit (Qiagen, Hilden, Germany). Paired-end sequencing (2 × 150 bp) of DNA libraries was done using an Illumina MiSeq or NextSeq100 platform (Illumina, San Diego, CA, USA). For the bioinformatic analysis, we used IMMense, an in-house developed pipeline based on Nextflow (25). Trimmomatic (version 0.39) was used to filter and trim raw sequencing data (26). Reads were assembled using Unicycler v0.4.8 (27). Genome assemblies were typed in Ridom Seqsphere+ v10.0.3 by multi-locus sequence typing (MLST). Detection of β-lactam resistance genes. Plasmid β-lactamase genes were identified using NCBI AMRFinder+ on the unicycler assemblies (28). All genome sequences generated at IMM, UZH, (CRPAZU001 through CRPAZU102, CRPAZU124, and CRPAZU125) were submitted to the ENA (https://www.ebi.ac.uk/ena/browser) under project number PRJEB82838.

## RESULTS

### Genetic analysis of bacterial strains

One hundred and thirty-six CRPA strains exhibited significant genetic diversity, representing 54 different MLST sequence types (STs, see Table S1). The most prevalent types were ST235 (*n* = 25) and ST111 (*n* = 12) (Table S1). Class B MBLs were detected in 69 CRPA isolates, with VIM being the most common MBL type (*n* = 38). Among these, the variants VIM-2 (*n* = 17) and VIM-4 (*n* = 13) were the most frequently observed. IMP was the second most prevalent MBL type (*n* = 17), with IMP-1 (*n* = 12) as the predominant variant. NDM-1 was identified in eight clinical CRPA isolates, while a combination of two MBL genes was found in three isolates (2× NDM-1/IMP-1 and 1× NDM-1/VIM-2). In one case, a combination of an MBL with an OXA-48-like carbapenemase (IMP-26/OXA-181) and with a class A GES-5 carbapenemase was detected. Less common MBL-encoding genes, such as *bla*_AIM-1_ and *bla*_SPM-1_, and class-A-carbapenemases-genes including *bla*_GES-5_ and a *bla*_KPC-2_ were found in one isolate each. Overall, one or more carbapenemase genes were identified in 71 (61.2%) CRPA strains. In eight of these cases, an ESBL was also detected (1× GES-7, 2× SHV, 1× VEB-9, 1× VEB-14, and 3× PME-1). Furthermore, ESBL-encoding genes were found in 12 non-CP-CRPA isolates (5× *bla*_GES_, 4× *bla*_VEB_, and 3× *bla*_PER_). Based on the detected resistance mechanisms, the CRPA isolates were categorized as follows: MBL (*n* = 60), MBL + GES-carba (*n* = 1), MBL + OXA-48 + ESBL (*n* = 1), MBL ± ESBL (*n* = 7), GES-carba (*n* = 1), KPC (*n* = 1), ESBL (*n* = 12), and negative (−, *n* = 53).

### *In vitro* susceptibility testing

#### Distributions of the growth inhibition zones of classic antibiotics

The susceptibility to standard antibiotics was determined using the DD assay. Based on the growth inhibition zone diameters and EUCAST clinical breakpoints (CBPs), all isolates were categorized as resistant to ceftazidime and cefepime, with the majority also resistant to imipenem (135/136, 99.3%) and/or non-susceptible to meropenem (116/136, 85.3%) (Fig. S1; Table S5). The vast majority were classified as resistant to piperacillin-tazobactam (133/136, 98.8%). No significant difference in susceptibility profiles was observed between CP/ESBL-CRPA and non-CP/ESBL-CRPA isolates.

Interestingly, although many isolates were categorized as resistant to aztreonam (91/136, 66.9%), the MBL-producing *P. aeruginosa* isolates exhibited, on average, larger growth inhibition zones compared to the ESBL producers and AmpC hyperproducers, consistent with the inability of class B MBLs to hydrolyze monobactams (Table S1). Most CRPA isolates were resistant to ciprofloxacin (111/136, 81.6%) and levofloxacin (117/136, 86%). Notably, CP/ESBL-CRPA isolates exhibited higher resistance to quinolones, with many showing high-level resistance (no growth inhibition = 6 mm, see Table S1). Similarly, though to a lesser extent, most isolates were resistant to amikacin (85/136, 62.5%) and tobramycin (98/136, 72%), with carbapenemase/ESBL producers showing significantly reduced susceptibility.

#### Distributions of the MICs and growth inhibition zones of the novel BL-BLI combinations

The susceptibilities to ceftolozane-tazobactam, ceftazidime-avibactam, meropenem-vaborbactam, cefepime-enmetazobactam, and imipenem-relebactam were tested using both DD and E-test methods (Table S6). Susceptibility to aztreonam-avibactam was assessed only by E-test, as commercially available discs were not yet available. The two methods yielded congruent results for ceftolozane-tazobactam, ceftazidime-avibactam, and meropenem-vaborbactam, with all BL-BLI combinations showing categorical agreement (CA) ranging from 89.7% to 93.8%, and classification errors occurring primarily with values near the CBPs (see Fig. S2). For imipenem-relebactam, the agreement between E-test and DD was less pronounced (CA = 80.1%), and a higher rate of very major errors (vMEs) was observed (17.6%), though these were mostly close to the CBP. For cefepime-enmetazobactam, despite showing a CA of 88.2%, the correlation between the two methods was notably weak. While DD revealed a broad range of growth inhibition zones, high MICs were consistently detected in nearly all CRPA isolates by E-test.

**Fig 1:**
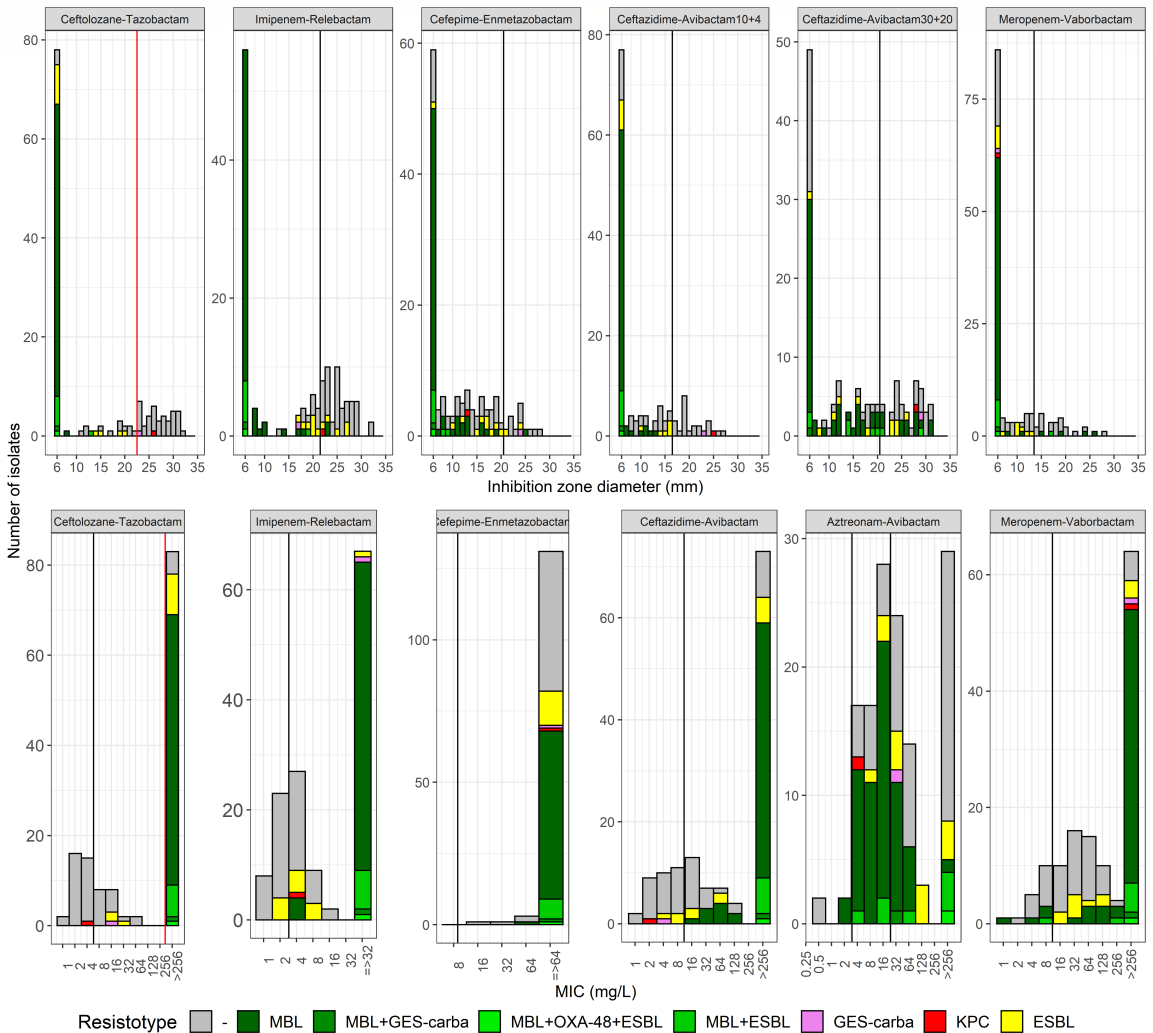
Distribution of disc diffusion growth inhibition zone diameters and minimal inhibitory concentrations (MICs). DD growth inhibition zone diameters (top) and MICs determined by E-test (bottom) of ceftolozane-tazobactam, imipenem-relebactam, cefepime-enmetazobactam, ceftazidime-avibactam, aztreonam-avibactam, and meropenem-vaborbactam are shown. The vertical black lines denote the EUCAST clinical breakpoints, while the vertical red lines define the proposed screening cut-offs for screening of carbapenemase(s)/ESBLs. Bacterial isolates are categorized according to their carbapenem-resistance marker(s). ESBL, extended-spectrum-β-lactamase; MBL, metallo-β-lactamase.

CP/ESBL-CRPA strains exhibited significantly smaller growth inhibition zones and higher MICs compared to AmpC hyperproducers for ceftolozane-tazobactam, ceftazidime-tazobactam, and meropenem-vaborbactam (Fig. 1). Cefepime-enmetazobactam showed a similar trend, though less pronounced. For imipenem-relebactam, while MBL-producing strains displayed significantly smaller growth inhibition zones and higher MICs, isolates producing Class-A-β-lactamases (CABL) including GES-5, KPC, and ESBL had values comparable to those of AmpC hyperproducers. Notably, MBL-, CABL-, and AmpC-hyperproducing CRPA strains showed similar distributions of growth inhibition zones and MICs for aztreonam-avibactam. Interestingly, CABL producers exhibited high-level resistance (MIC >256 mg/L, with no detectable growth inhibition zone) to meropenem-vaborbactam, while displaying variable susceptibility to other BL-BLI combinations.

#### Development of a phenotypic algorithm for detection of MBL and ESBL in CRPA

Screening for MBL and ESBL producers. The analysis of the distributions of growth inhibition diameters and MICs for BL-BLI combinations showed that ceftolozane-tazobactam provides the best separation between MBL and ESBL producers from AmpC hyperproducers (Fig. 1). Using <23 mm (A) and >256 mg/L (B) as screening cut-offs successfully included all MBL producers (Table 1). While the disk diffusion cut-off also captured all 12 ESBL and 14 out of 53 AmpC hyperproducers, the MIC-based cut-off included 9 out of 12 ESBL and 9 out of 53 AmpC hyperproducers. Notably, neither the class-A carbapenemase GES-5 nor KPC-2 producers screened positive using both DD and E-test methods.

**TABLE 1:** Performances of the diagnostic algorithm^*a*^

Testing step	Screening for carbapenemases and ESBLs (KPC excluded)		Confirmatory testing for MBL			Confirmatory testing for class A ESBL producers	
Method	DD	E-test	DD	LFIA	LFIA + DD	DD	DD + double disc synergy test
Cut-off	C-T <23 mm	C/T >256 mg/L	C-T <23 mm, I-R <16 mm	C-T <23 mm, Carba-5 positive	C-T <23 mm, Carba-5 positive, I-R <16 mm	C-T <23 mm, I-R >15 mm, C-E >14 mm	C-T <23 mm, I-R >15 mm, C-E >14 mm, synergy C-E/FEP
Sensitivity for MBL	69/69 (100%)	69/69 (100%)	64/69 (92.8%)	67/69 (97%)	69/69 (100%)		
Sensitivity for class-A carbapenemases (GES-5, KPC-2)	0/2 (0%)	0/2 (0%)					
Sensitivity for class-A ESBL	12/12 (100%)	9/12 (75%)				9/12 (75%)	6/9 (66.6%)
Specificity	14/53 (73.6%)	5/53 (90.6%)	0/26 (100%)	0/26 (100%)	0/26 (100%)	3/14 (78.6%)	0/3 (100%)

^*a*^ ESBL, extended-spectrum β-lactamase; MBL, metallo β-lactamase; C-T, ceftolozane-tazobactam; I-R, imipenem-relebactam; C-E, cefepime-enmetazobactam.

#### Phenotypic detection of MBL and ESBL producers

**Fig 2:**
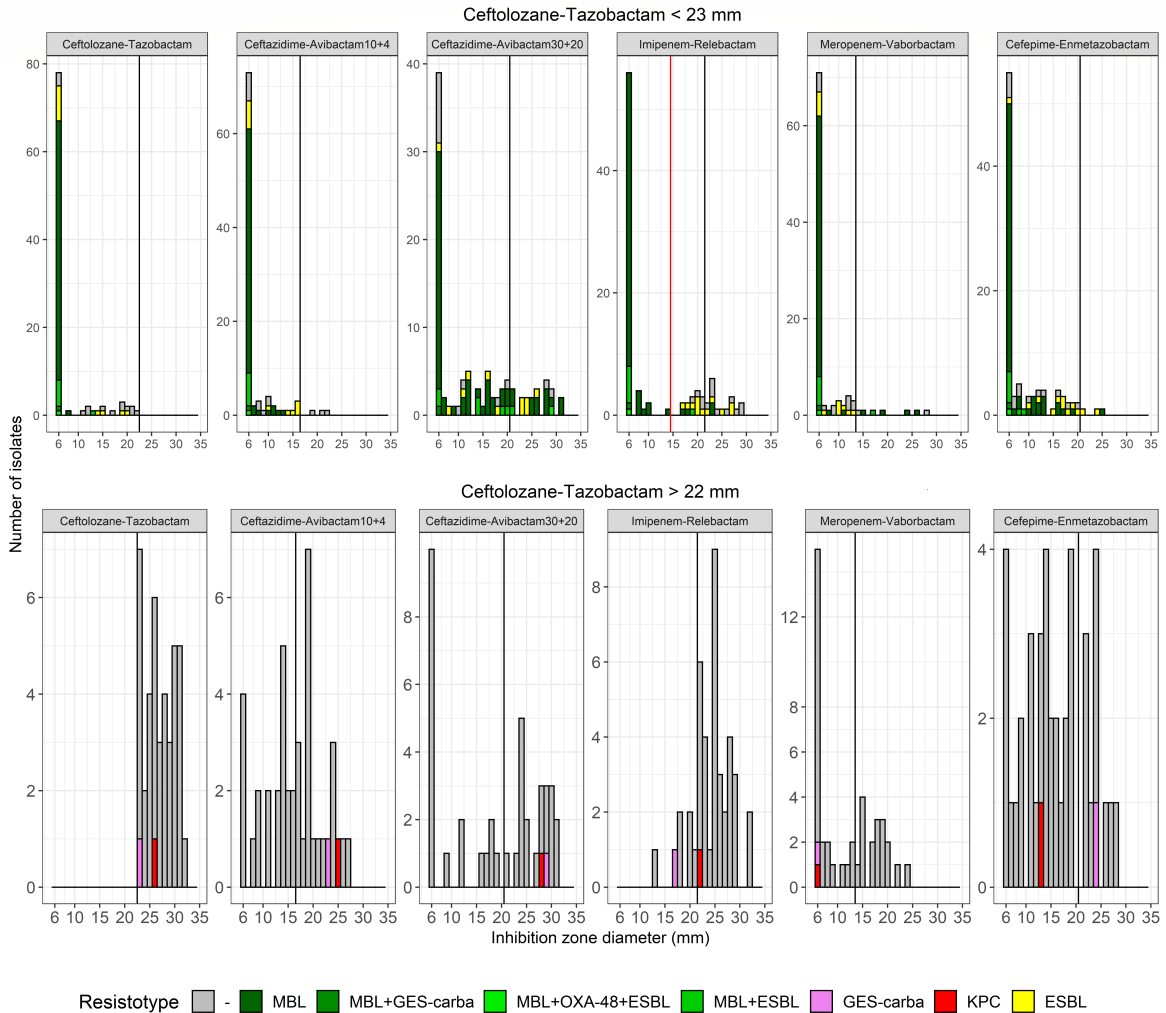
Distribution of disc diffusion growth inhibition zone diameters of isolates suspected of carbapenemase(s)/ESBL production (top) and screened negative (bottom). Isolates were grouped according to the carbapenem-resistance marker(s). The vertical black lines denote the EUCAST clinical breakpoints, while the vertical red lines define the proposed screening cut-off for confirmation of MBL production. ESBL, extended-spectrum-β-lactamase; MBL, metallo-β-lactamase.

**Fig 3:**
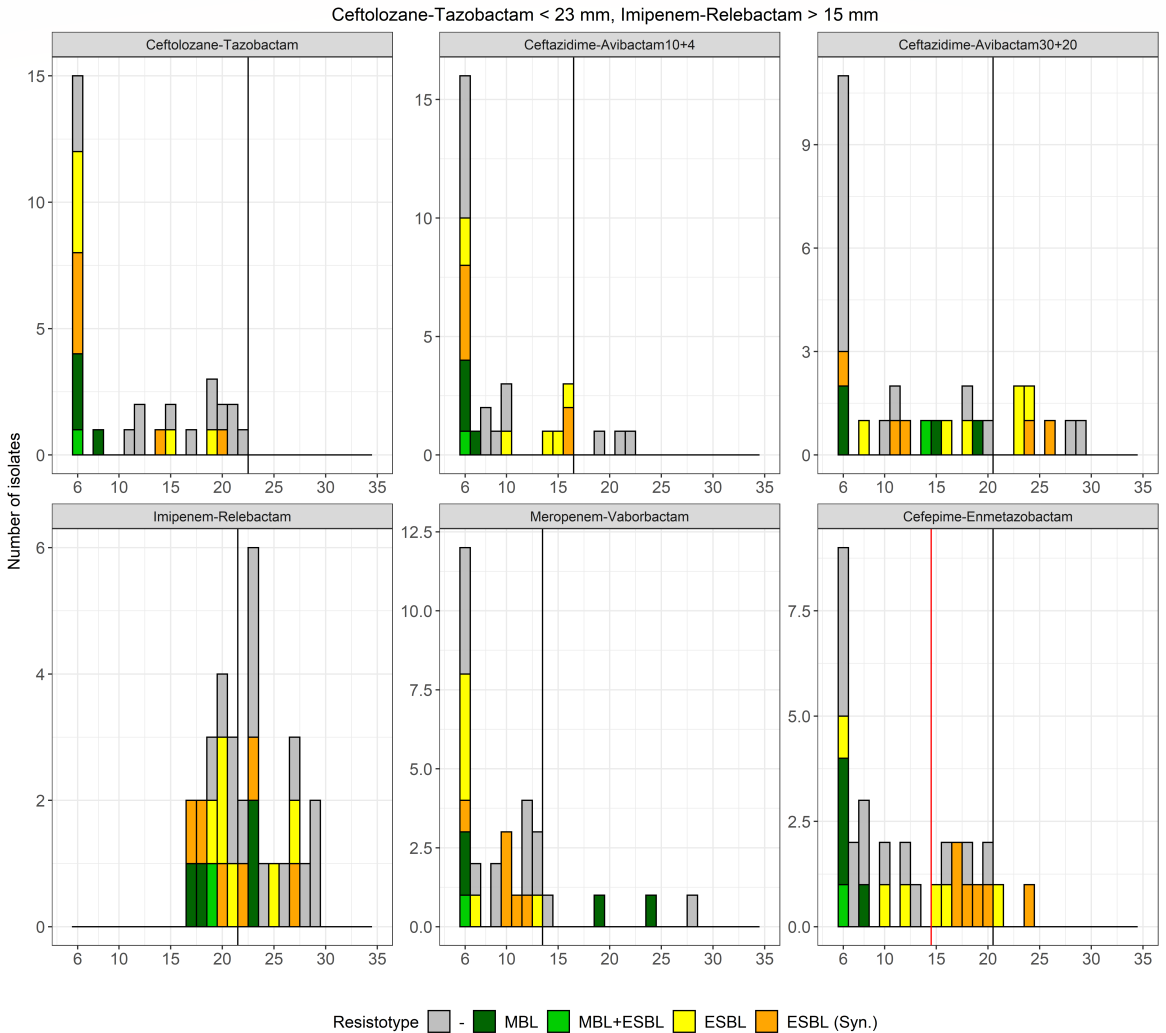
Distribution of disc diffusion growth inhibition zone diameters of isolates screened negative for carbapenemase production. Isolates were grouped according to the carbapenem-resistance marker(s). ESBL producers that tested positive by DDST are highlighted in orange. The vertical black lines denote the EUCAST clinical breakpoints, while the vertical red lines define the proposed screening cut-off for confirmation of ESBL production. ESBL, extended-spectrum-β-lactamase; MBL, metallo-β-lactamase.

**Fig 4:**
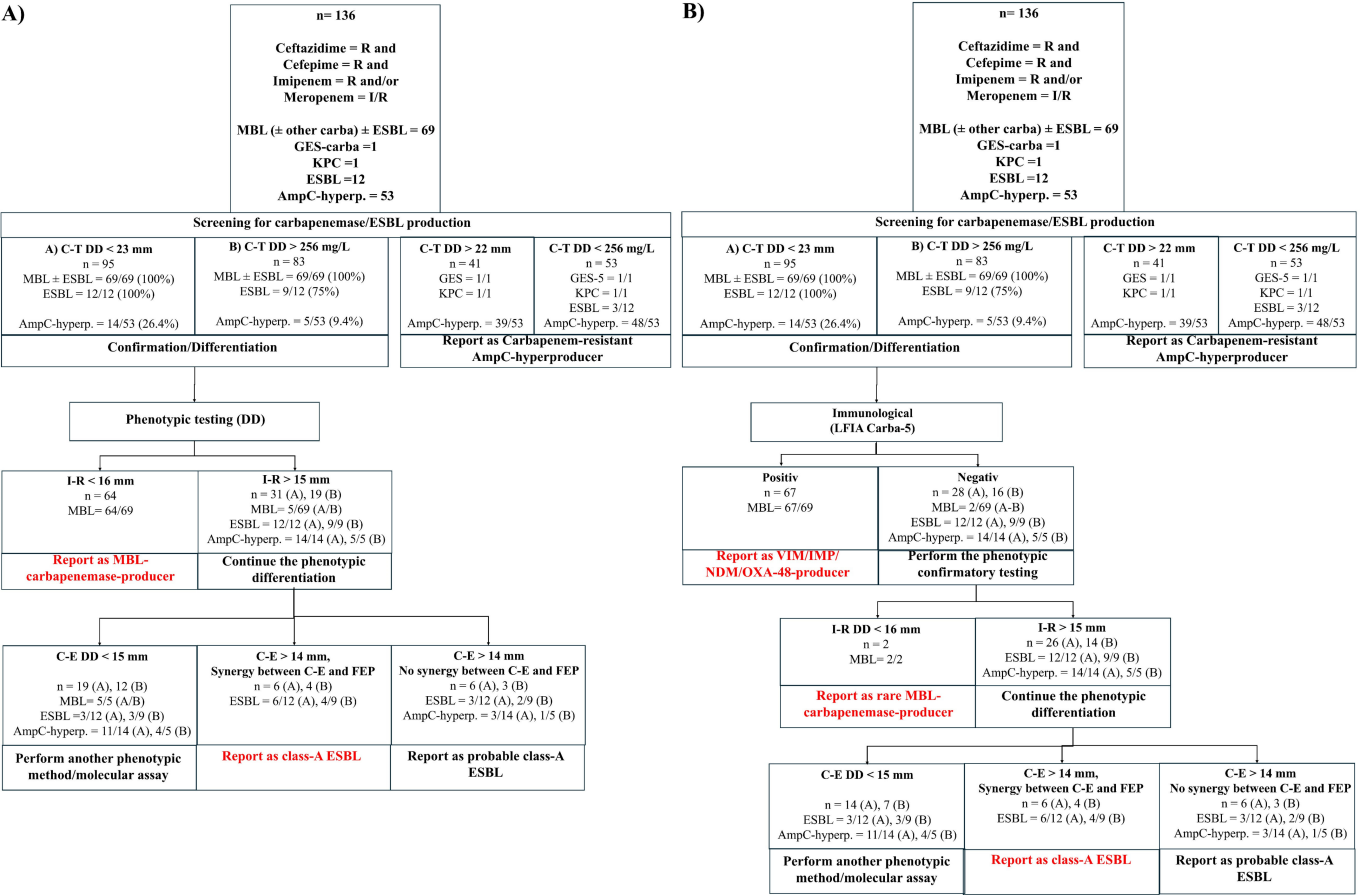
Phenotype- (**A**) and phenotype-/LFIA- (**B**) based diagnostic algorithm for screening, detection, and differentiation of MBL and class-A-β-lactamases in CRPA.

The analysis of growth inhibition zones and MIC values of CRPA isolates suspected of producing MBL-ESBL based on the ceftolozane-tazobactam cut-off demonstrated that imipenem-relebactam provides excellent separation between MBL producers and ESBL or AmpC hyperproducers (Fig. 2). Given that DD yielded significantly better performance than the E-test (which did not allow further differentiation of β-lactamase production, see Fig. S3), only DD data were included in the algorithm. Using a cut-off of <16 mm, 64 out of 69 MBL producers (92.8%) could be phenotypically confirmed (Table 1). The remaining isolates (5/69 MBL, 12/12 [A] or 9/9 [B] ESBL, and 14/14 [A] or 5/5 [B] AmpC hyperproducers) exhibited growth inhibition zones for imipenem-relebactam >15 mm and were further investigated. The analysis of the growth inhibition zone distributions of these latter isolates indicated that cefepime-enmetazobactam could effectively separate ESBL producers from the remaining isolates (Fig. 3). Using a cut-off of >14 mm, 9 out of 12 (A) and 6 out of 9 (B) ESBL producers could be phenotypically confirmed (Fig. 4). Three out of 14 (A) and 1 out of 5 (B) AmpC hyperproducers also exhibited a growth inhibition zone of >14 mm. The remaining isolates with a value lower than 15 mm included the 5 out of 5 MBL (A/B), 3 out of 12 (A) and 3 out of 9 (B) ESBL producers, and 11 out of 14 (A) and 4 out of 5 (B) AmpC hyperproducers.

#### Immunological and phenotypic confirmation/discrimination of MBL and ESBL producers

CRPA isolates suspected of being MBL-ESBL producers (exhibiting ceftolozane-tazobactam mm <23 mm or >245 mg/L) were subjected to immunological detection of MBLs using the LFIA Carba-5, which successfully detected all VIM, IMP, NDM, and OXA-48-only or multiple producers (*n* = 67/69 MBL, see Table 1). As expected, SPM-1 and AIM-1 were not detected. Additionally, Carba-5 produced negative results for the ESBL and AmpC hyperproducers that had screened positive with ceftolozane-tazobactam. Using an imipenem-relebactam cut-off of <16 mm, both SPM-1 and AIM-1 producers could be then phenotypically confirmed. Like the previous algorithm based solely on phenotypic data, the remaining isolates showing a growth inhibition zone for imipenem >15 mm were further investigated using cefepime-enmetazobactam. Using a >14 mm cut-off, 9 out of 12 (A) and 6 out of 9 (B) ESBL producers were confirmed, while some AmpC hyperproducers (3/14 [B] and 1/5 [B]) also showed inhibition zones >14 mm. Three out of 12 (A) and 3 out of 9 (B) ESBL producers, along with 11 out of 14 (A) and 4 out of 5 (B) AmpC producers, had cefepime-enmetazobactam inhibition zones <15 mm.

#### ESBL detection by synergy testing

To improve the performance of the diagnostic algorithm in detecting class A ESBLs, we used DDST on isolates suspected of being ESBL producers (screened positive with ceftolozane-tazobactam and exhibiting imipenem-relebactam growth inhibition zones >15 mm), following approaches established in previous studies (17, 29, 30). To keep the algorithm simple and limit the number of tested compounds, DDST was performed between the two BL-BLI combinations already used for confirmatory testing—imipenem-relebactam and cefepime-enmetazobactam—along with third-generation cephalosporins ceftazidime and cefepime. These have been previously shown to be the best substrates for detecting ESBLs most found in *P. aeruginosa*. Different distances between the discs (12, 15, and 20 mm) were tested. The most prominent inhibition zones between the BL-BLI combinations were observed when the discs were placed 12 mm apart. Cefepime was found to be the most sensitive substrate for detecting ESBLs (data not shown). Furthermore, cefepime-enmetazobactam proved to be the most specific BL-BLI combination for ESBL detection (19/19, 100%), although it was less sensitive than imipenem-relebactam (6/12, 50% vs 10/12, 83.3%, respectively, see Table S3). However, imipenem-relebactam showed lower specificity (15/19, 78.9%). Interestingly, synergy between cefepime-enmetazobactam and cefepime was observed in isolates with a cefepime-enmetazobactam inhibition zone larger than 14 mm (6/9, 66.6%). Based on these findings, DDST between cefepime-enmetazobactam and cefepime with a 12 mm disc distance was incorporated into the diagnostic algorithm. For isolates that tested positive with ceftolozane-tazobactam (inhibition zone <23 mm or MIC >256 mg/L) and displayed inhibition zones larger than 15 mm for imipenem-relebactam and larger than 14 mm for cefepime-enmetazobactam, if an enhanced zone of inhibition is detected, the isolate can be reported as an ESBL producer; otherwise, it may be reported as a probable ESBL producer.

### Performance of the algorithms

#### Screening for carbapenemase and ESBL producers

Overall, applying a CBP cut-off of 23 mm and an MIC of >256 mg/L as thresholds resulted in the successful screening of all 69 MBL producers included in the study (100% sensitivity, see Table 1). However, while the 23 mm cut-off allowed for the screening of all 12 ESBL producers (100% sensitivity), the >256 mg/L threshold only detected 9 out of 12 (75%). Notably, neither GES-5 nor KPC class A carbapenemase producers were detected with ceftolozane-tazobactam using either the DD method or E-test. Additionally, the 23 mm cut-off also incorrectly included 14 out of 53 AmpC hyperproducers (specificity of 73.6%), whereas the >256 mg/L cut-off only included 5 out of 53 (specificity of 90.6%, see Table 1).

#### Confirmatory testing for MBL

The cut-off of <16 mm for imipenem-relebactam demonstrated very good performance in detecting MBL producers, identifying 64 out of 69 cases and excluding all ESBL and AmpC hyperproducers (92.8% sensitivity and 100% specificity, see Table 1). Using the LFIA Carba-5 assay detected 67 out of 69 MBL producers, with no false positives (97% sensitivity, 100% specificity). Combining both methods—first performing the LFIA and then applying the imipenem-relebactam cut-off of 16 mm—successfully identified all 69 MBL producers (100% sensitivity) with no false positives (100% specificity).

#### Confirmatory testing for class A ESBLs

The cut-off of >14 mm for cefepime-enmetazobactam identified 9 out of 12 ESBL producers (75% sensitivity) and 3 out of 14 AmpC hyperproducers (78.6% specificity, see Table 1). Moreover, only six out of nine ESBL producers showed a clear enhancement in growth inhibition between cefepime-enmetazobactam and cefepime. Considering this as evidence of ESBL production, the combination of the cefepime-enmetazobactam cut-off >14 mm and synergy testing detected 6 out of 12 ESBL producers (50% sensitivity) with no false positives (100% specificity).

## DISCUSSION

Several phenotypic and molecular methods for detecting carbapenemases in CRPA have been reported, each with varying performance and limitations. However, few studies have focused on identifying which CRPA strains should undergo carbapenemase/ESBL testing. In a recent multicenter, prospective study by Gill et al., a phenotypic algorithm was validated to guide carbapenemase detection in CRPA. Using MIC data from 807 CRPA isolates, they demonstrated that incorporating ceftolozane-tazobactam nonsusceptibility into previous screening criteria (resistance to imipenem or meropenem, plus nonsusceptibility to ceftazidime and cefepime) significantly increased the specificity without affecting the sensitivity of the phenotypic algorithm (31). In our study, we show that both DD and E-test values for ceftolozane-tazobactam alone can effectively distinguish MBL and ESBL producers from AmpC hyperproducers in CRPA exhibiting resistance to ceftazidime, cefepime, imipenem, and/or meropenem. Furthermore, Gill et al. found that, among the 316 CRPA isolates that screened negative, only 4 were confirmed to produce carbapenemases genotypically (VIM = 2, KPC = 1, and GES = 1). In comparison, in our study, ceftolozane-tazobactam screening cut-offs of <23 mm missed the single class-A-carbapenemase KPC and a few GES producers, while none of the MBL producers (mostly VIM producers) screened negative. This result underscores the robustness of ceftolozane-tazobactam in screening for MBL producers, due to the ability of MBL to hydrolyze ceftolozane *in vitro* without inhibition by tazobactam (32). As noted in the study by Gill et al., ceftolozane-tazobactam was not fully effective against certain GES β-lactamase producers. This could be attributed to the variable production and/or hydrolytic capabilities within this diverse enzyme class (33). Ceftolozane-tazobactam's excellent ability to differentiate between MBL-ESBL producers and AmpC hyperproducers is largely due to its stability against the latter (34). Similarly, in our study, although all class-A-ESBL producers screened positive, they displayed variable growth inhibition zones within the resistance range. Ceftolozane-tazobactam stands out as the optimal candidate for screening carbapenemase production in *P. aeruginosa*, particularly in regions with a high prevalence of MBL producers. Laboratories performing AST by DD may already include ceftolozane-tazobactam in their testing panels for *P. aeruginosa*, thereby facilitating rapid and timely detection of carbapenemase producers and reducing unnecessary tests. This is especially advantageous in low-income countries, where DD is the predominant AST method due to its affordability and where data on the prevalence of CP CRPA is limited.

Imipenem-relebactam exhibited a similar profile to ceftolozane-tazobactam. While demonstrating high-level resistance—evidenced by either no growth inhibition zones or high MICs—against MBL-producing strains (though not across all strains), this BL-BLI combination retained some activity against both ESBL and AmpC hyperproducers. This can be attributed to relebactam’s excellent ability to inhibit class A and class C enzymes, though it lacks activity against MBLs (35). This characteristic has been utilized to establish a DD cut-off for confirming MBL production in CRPA strains tested positive when screened with ceftolozane-tazobactam. Notably, in this context, DD showed enhanced performance compared to the E-test method, possibly due to the high amount of inhibitor used in the disc (35 µg) compared to the lower concentration evenly applied throughout the E-test strip (4 µg/mL).

The strong inhibitory activity of the novel BLI enmetazobactam against ESBLs (36) was used to further distinguish between MBL and AmpC producers (against which enmetazobactam is inactive) from ESBL producers (which retained some activity). A cut-off of >14 mm reliably detected ESBLs in CRPA isolates that tested positive with ceftolozane-tazobactam but negative with imipenem-relebactam. For the remaining CRPA isolates, where the resistance mechanisms were unclear, additional or alternative phenotypic or molecular methods are needed. DDST between cefepime-enmetazobactam and cefepime, with a 12 mm disc distance, confirmed 6 out of 12 ESBL producers exhibiting growth inhibition zones for ceftolozane-tazobactam <23 mm and for imipenem-relebactam >15 mm. All six positive isolates showed a cefepime-enmetazobactam growth inhibition zone greater than 14 mm, supporting the hypothesis that AmpC hyperproduction (which enmetazobactam does not inhibit) may interfere and potentially lead to false negative results. The use of the AmpC inhibitor cloxacillin in the disc or plate may potentially enhance the performance of DDST in detecting class A ESBLs. Interestingly, cefepime demonstrated greater sensitivity than ceftazidime, consistent with the notion that cefepime is a good substrate for ESBLs but a weaker one for AmpC (37). Additionally, we observed that disc distance significantly influenced the detection of synergy phenomena. Based on these findings, further studies with larger collections comprising more ESBL types and variants are needed to optimize this method.

In cases of suspected carbapenemase or ESBL production, a phenotypic confirmatory/discriminatory test involving imipenem-relebactam, cefepime/enmetazobactam, and cefepime DD could be easily implemented. Notably, most commercial molecular methods and LFIA currently do not detect rare ESBLs in *Enterobacterales* (these primarily include the detection of CTX-M-type enzymes), such as GES and VEB enzymes, which are more commonly found in non-fermenters like *P. aeruginosa*. This makes the diagnostic algorithm particularly valuable for identifying such enzymes.

Implementing LFIA into the diagnostic algorithm would enhance and expedite MBL detection, particularly in regions with a high prevalence of VIM-, IMP-, and NDM-type MBLs, which are specifically targeted by the LFIA. Additionally, other commercial LFIA tests for detecting ESBLs, such as the one targeting GES, which is currently in development (38), may potentially be integrated into this diagnostic algorithm.

The major strength of this study is the inclusion of 136 genotypically characterized and geographically diverse CRPA exhibiting high-level resistance to classic β-lactam antibiotics and including a high number of carbapenemase/ESBL-negative CRPA. However, this study presents some limitations. First, the diagnostic algorithm was developed and validated using a collection of CRPA strains that predominantly reflect the prevalence in Europe, which mainly includes MBL and specifically VIM producers (19), despite the inclusion of strains from Pakistan. Regions with different epidemiological profiles, such as those with high prevalence of GES-carbapenemase or KPC producers, may need to adapt the algorithm accordingly. Notably, the single GES and KPC producers both exhibited high-level resistance to meropenem-vaborbactam while showing variable susceptibility to other BL-BLI combinations. Therefore, meropenem-vaborbactam might be considered for screening GES-carbapenemase and KPC producers, with confirmation potentially achieved using standard LFIA or molecular methods. Second, the number of ESBL-only producers in the study was limited (*n* = 12). Due to substrate variability of class A ESBL found in *P. aeruginosa*, further studies with larger collections including more and diverse ESBL types and variants are needed to assess the robustness of the algorithm in detecting and discriminating ESBL production.

Overall, this study presents a simple and cost-effective phenotypic algorithm for the screening, confirmation, and discrimination of MBL- and ESBL-producing as well as from AmpC-hyperproducing CRPA strains. This diagnostic algorithm can be implemented in any laboratory and has the potential to enhance global understanding of the prevalence of plasmid-based MBL and ESBL production in CRPA.

## SUPPLEMENTAL MATERIAL

Supplemental material spectrum.03196-24-s0001.docx Fig. S1 to S3; Tables S1 to S6.

ASM does not own the copyrights to Supplemental Material that may be linked to, or accessed through, an article. The authors have granted ASM a non-exclusive, world-wide license to publish the Supplemental Material files. Please contact the corresponding author directly for reuse.
