# Supplementary material for: Diagnostic algorithm for the detection of carbapenemases and extended-spectrum β-lactamases in carbapenem-resistant Pseudomonas aeruginosa
Source: Microbiol Spectr. 2025 Apr 16;13(6):e03196-24. doi: 10.1128/spectrum.03196-24 (PMC12131727; doi:10.1128/spectrum.03196-24)
Supplement: Supplemental material — Fig. S1 to S3; Tables S1 to S6. [file spectrum.03196-24-s0001.docx]

**Supplementary data**

**Supplementary figures**

**Figure S1. Distribution of disc diffusion growth inhibition zone diameters of classic antibiotics. Isolates were grouped according to the carbapenem-resistance marker(s).** The vertical black lines denote the EUCAST clinical breakpoints.


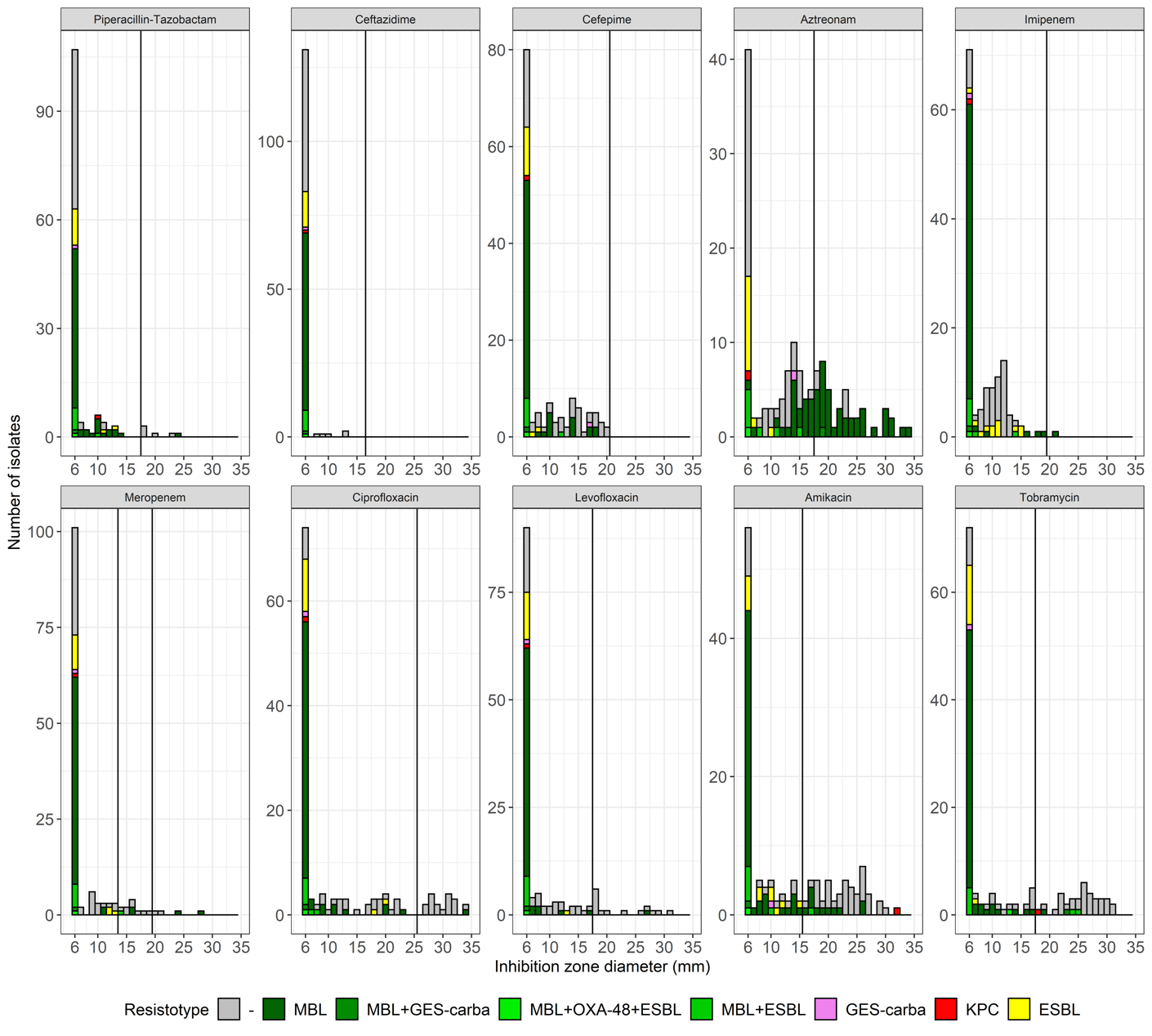
MBL, metallo-β-lactamase; ESBL, extended-spectrum-β-lactamase.

**
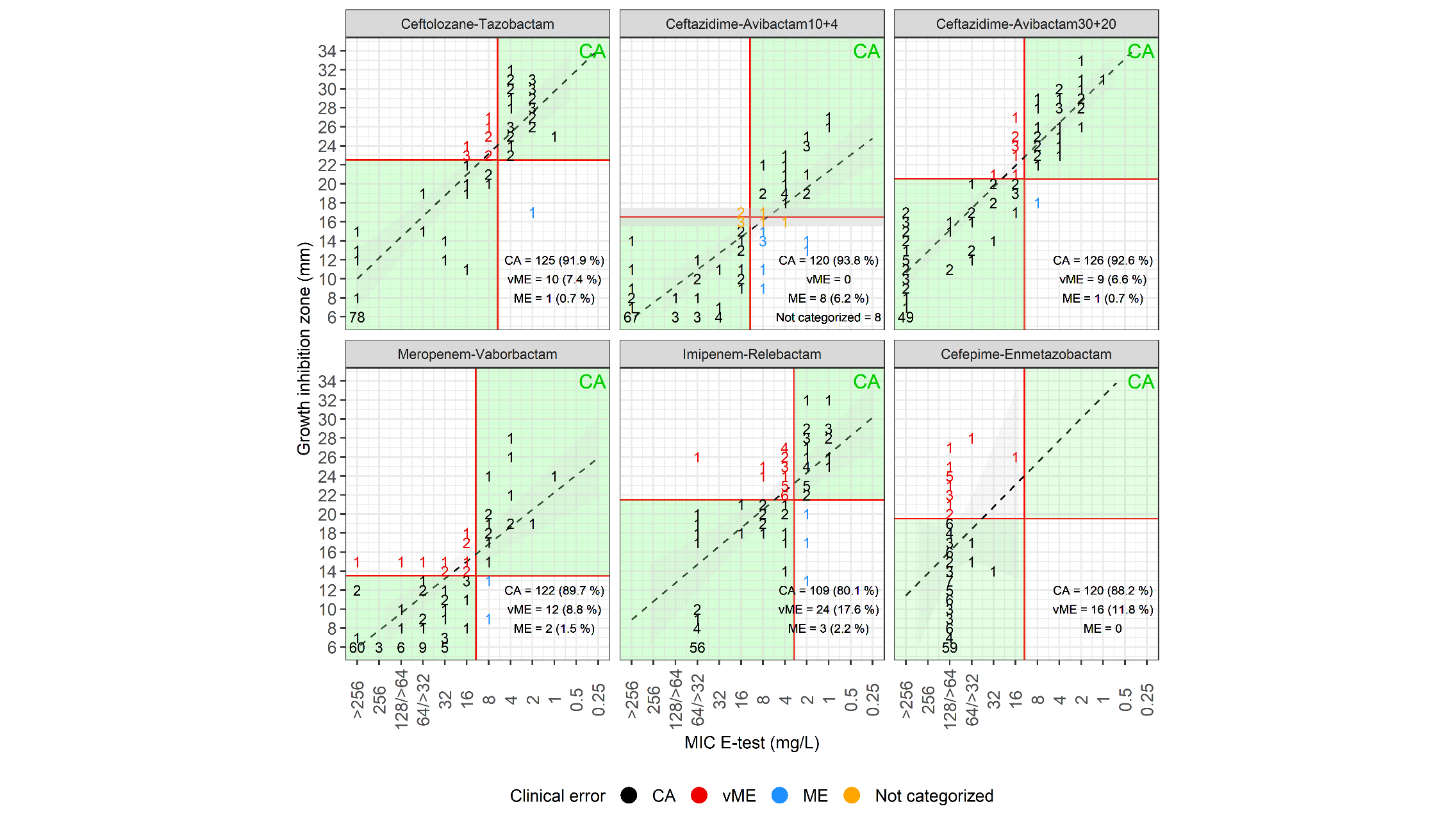
Figure S2. Disc diffusion versus E-test.** Disc diffusion growth inhibition zone diameters versus MICs determined by E-test. MICs are on the x axis and zone diameters on the y axis. Isolates were categorized according to the E-test MICs and EUCAST CBPs. MICs/zone diameters were classified as categorical agreement in black, very major error in red and major error in blue. Isolates depicted in orange fell within the area of technical uncertainty (ATU) and were not categorized. The red continous lines denote the EUCAST CBPs. The black dashed lines denote the regression lines. The green areas denote zones of congruence between the two methods, while the gray areas denote the ATU.

**Figure S3. Distribution of MICs of isolates suspected of carbapenemase(s)/ESBL production (MIC for ceftolozane-tazobactam > 256 mg/L).** Isolates were grouped according to the carbapenem-resistance marker(s). The vertical black lines denote the EUCAST clinical breakpoints, while the vertical red lines define the proposed screening cut-off for confirmation of MBL production.


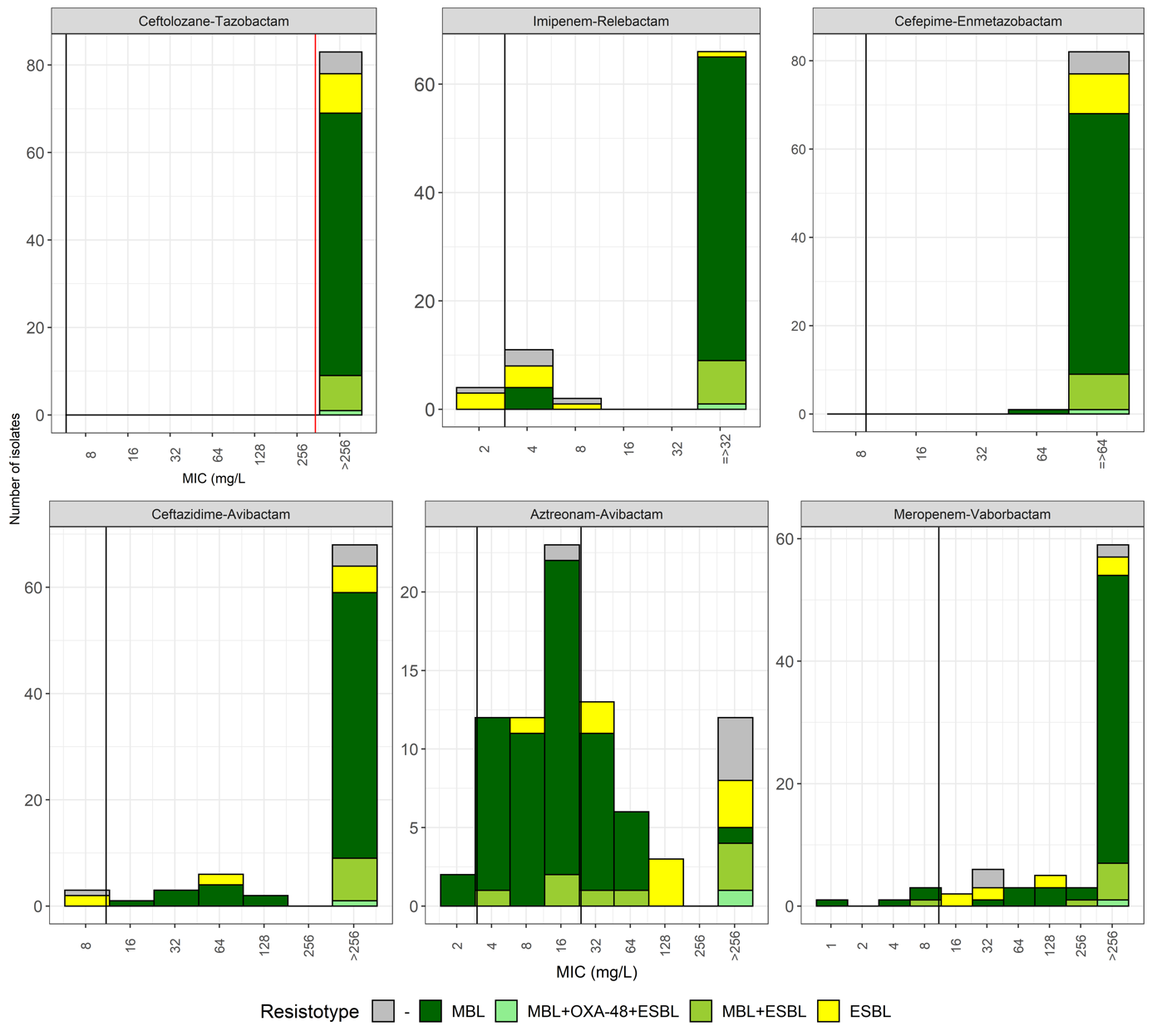
MBL, metallo-β-lactamase; ESBL, extended-spectrum-β-lactamase.

**Supplementary Tables**

**Table S1 Genetic features of the CRPA included in the study and results of Carba-5**

| **Study number** | **Sequence type** | **Carba-5** | **Carbapenemase** | **ESBL** | **Group** | **Intrinsic AmpC (PDC)** | **Intrinsic oxacillinase** | **Others** |
| --- | --- | --- | --- | --- | --- | --- | --- | --- |
| CRPAZU0001 | 253 | NEG | - | - | - | PDC-33 | OXA-488 | - |
| CRPAZU002 | 664 | NEG | - | GES-12 | ESBL | PDC-98 | OXA-50 | - |
| CRPAZU003 | 2572 | NEG | - | - | - | PDC-374 | OXA-488 | - |
| CRPAZU004 | 235 | VIM | VIM-4 | - | MBL | PDC-35 | OXA-488 | OXA-35/CARB-2 |
| CRPAZU005 | 941 | NEG | - | - | - | PDC-374 | OXA-396 | - |
| CRPAZU006 | 485 | NEG | - | - | - | PDC-6 | OXA-50 | - |
| CRPAZU007 | 245 | IMP | IMP-18 | - | MBL | PDC-5 | OXA-494 | OXA-10 |
| CRPAZU008 | 282 | VIM | VIM-4 | - | MBL | PDC-103 | OXA-50 | CARB-2 |
| CRPAZU009 | 357 | IMP | IMP-7 | - | MBL | PDC-11 | OXA-846 | OXA-2 |
| CRPAZU010 | 4936 | IMP | IMP-1 | - | MBL | PDC-35 | OXA-50 | OXA-10/PAC-1 |
| CRPAZU011 | 285 | NEG | - | - | - | PDC-1 | OXA-494 | - |
| CRPAZU012 | 385 | NEG | - | - | - | PDC-5 | OXA-396 | - |
| CRPAZU013 | 111 | VIM | VIM-2 | - | MBL | PDC-3 | OXA-395 | - |
| CRPAZU014 | 316 | NEG | - | - | - | PDC-36 | OXA-395 | OXA-10 |
| CRPAZU015 | 316 | NEG | - | - | - | PDC-36 | OXA-395 | OXA-10 |
| CRPAZU016 | 3416 | NEG | - | - | - | PDC-23 | OXA-848 | - |
| CRPAZU017 | 4936 | IMP | IMP-1 | - | MBL | PDC-35 | OXA-50 | OXA-10/PAC-1 |
| CRPAZU018 | 316 | NEG | - | - | - | PDC-36 | OXA-395 | OXA-10 |
| CRPAZU019 | 357 | IMP + OXA-48 | OXA-181/IMP-26 | VEB-9 | MBL+OXA-48+ESBL | PDC-3 | OXA-486 | OXA-10 |
| CRPAZU020 | 3416 | NEG | - | - | - | PDC-23 | OXA-848 | - |
| CRPAZU021 | 111 | VIM | VIM-2 | - | MBL | PDC-3 | OXA-395 | - |
| CRPAZU022 | 2572 | NEG | - | - | - | PDC-3 | OXA-488 | - |
| CRPAZU023 | 941 | NEG | - | - | - | PDC-374 | OXA-396 | - |
| CRPAZU024 | 664 | NEG | - | GES-12 | ESBL | PDC-98 | OXA-50 | - |
| CRPAZU025 | 111 | VIM | VIM-4 | - | MBL | PDC-3 | OXA-395 | OXA-9/CARB-2 |
| CRPAZU026 | 189 | NEG | - | - | - | PDC-374 | OXA-905 | - |
| CRPAZU027 | 485 | NEG | - | - | - | PDC-374 | OXA-50 | - |
| CRPAZU028 | 235 | VIM | VIM-2 | - | MBL | PDC-35 | OXA-488 | - |
| CRPAZU029 | 111 | VIM | VIM-2 | - | MBL | PDC-3 | OXA-395 | - |
| CRPAZU030 | 1047 | IMP | IMP-1 | - | MBL | PDC-12 | OXA-488 | OXA-10 |
| CRPAZU031 | 111 | NEG | - | - | - | PDC-3 | OXA-395 | OXA-14/CARB-2 |
| CRPAZU032 | 155 | VIM | VIM-1 | GES-7 | MBL+ESBL | PDC-5 | OXA-396 | - |
| CRPAZU033 | 988 | NEG | - | - | - | PDC-23 | OXA-904 | - |
| CRPAZU034 | 274 | NEG | - | - | - | PDC-24 | OXA-486 | - |
| CRPAZU035 | 253 | NEG | - | - | - | PDC-34 | OXA-488 | - |
| CRPAZU036 | 111 | VIM | VIM-2 | - | MBL | PDC-3 | OXA-395 | - |
| CRPAZU037 | 4072 | NEG | - | - | - | PDC-374 | OXA-486 | - |
| CRPAZU038 | 235 | VIM | VIM-4 | - | MBL | PDC-35 | OXA-488 | OXA-35/CARB-2 |
| CRPAZU039 | 1420 | NEG | - | - | - | PDC-19a | OXA-395 | - |
| CRPAZU040 | 485 | NEG | - | - | - | PDC-6 | OXA-50 | - |
| CRPAZU041 | 235 | VIM | VIM-4 | - | MBL | PDC-35 | OXA-488 | CARB-2 |
| CRPAZU042 | 27 | NEG | - | - | - | PDC-15 | OXA-494 | - |
| CRPAZU043 | 1171 | NEG | - | - | - | PDC-97 | OXA-50 | - |
| CRPAZU044 | n.t. | NEG | - | - | - | PDC-432 | OXA-1124 | - |
| CRPAZU045 | 235 | NEG | - | VEB-16 | ESBL | PDC-35 | OXA-488 | OXA-10 |
| CRPAZU046 | 235 | VIM | VIM-4 | - | MBL | PDC-35 | OXA-488 | OXA-35/CARB-2 |
| CRPAZU047 | 111 | NEG | - | - | - | PDC-3 | OXA-395 | OXA-9 |
| CRPAZU048 | 357 | NEG | - | VEB-9 | ESBL | PDC-11 | OXA-846 | OXA-10 |
| CRPAZU049 | 235 | NEG | AIM-1 | SHV-5 | MBL+ESBL | PDC-35 | OXA-488 | - |
| CRPAZU050 | 235 | VIM | VIM-28 | SHV-2a | MBL+ESBL | PDC-35 | OXA-488 | - |
| CRPAZU051 | 111 | VIM | VIM-4 | - | MBL | PDC-3 | OXA-395 | OXA-9/CARBA-2 |
| CRPAZU052 | 111 | NEG | - | - | - | PDC-3 | OXA-395 | OXA-9 |
| CRPAZU053 | 235 | VIM | VIM-2 | - | MBL | PDC-35 | OXA-488 | - |
| CRPAZU054 | 235 | VIM | VIM-4 | - | MBL | PDC-35 | OXA-488 | OXA-35/CARB-2 |
| CRPAZU055 | 235 | VIM | VIM-4 | - | MBL | PDC-35 | OXA-488 | OXA-35/CARB-2 |
| CRPAZU056 | 175 | VIM | VIM-1 | - | MBL | PDC-1 | OXA-50 | - |
| CRPAZU057 | 235 | NEG | - | - | - | PDC-35 | OXA-488 | OXA-35/CARB-2 |
| CRPAZU058 | 164 | NEG | - | - | - | PDC-121 | OXA-486 | - |
| CRPAZU059 | 357 | NEG | - | VEB-9 | ESBL | PDC-11 | OXA-846 | OXA-10 |
| CRPAZU060 | 235 | VIM | VIM-4 | - | MBL | PDC-35 | OXA-488 | OXA-35/CARB-2 |
| CRPAZU061 | 235 | NEG | - | GES-52 | ESBL | PDC-35 | OXA-488 | - |
| CRPAZU062 | 635 | NEG | - | - | - | PDC-8 | OXA-395 | - |
| CRPAZU063 | 235 | VIM | VIM-2 | - | MBL | PDC-35 | OXA-488 | OXA-10 |
| CRPAZU064 | 316 | NEG | - | - | - | PDC-36 | OXA-395 | OXA-10 |
| CRPAZU065 | 316 | NEG | - | - | - | PDC-36 | OXA-395 | OXA-10 |
| CRPAZU066 | 111 | VIM | VIM-2 | - | MBL | PDC-3 | OXA-395 | - |
| CRPAZU067 | 235 | VIM+NDM | NDM-1/VIM-2 | - | MBL | PDC-35 | OXA-488 | - |
| CRPAZU068 | 2553 | IMP | IMP-1 | - | MBL | PDC-3 | OXA-486 | - |
| CRPAZU069 | 155 | NEG | - | - | - | PDC-5 | OXA-396 | - |
| CRPAZU070 | 266 | NEG | - | - | - | PDC-1 | OXA-486 | - |
| CRPAZU071 | 357 | VIM | VIM-5 | VEB-14 | MBL+ESBL | PDC-11 | OXA-846 | OXA-10 |
| CRPAZU072 | 1047 | IMP | IMP-1 | - | MBL | PDC-12 | OXA-488 | OXA-10 |
| CRPAZU073 | 235 | VIM | VIM-4 | - | MBL | PDC-35 | OXA-488 | CARB-2 |
| CRPAZU074 | 654 | VIM | VIM-2 | - | MBL | PDC-3 | OXA-396 | - |
| CRPAZU075 | 179 | NEG | - | PER-1 | ESBL | PDC-8 | OXA-396 | OXA-4 |
| CRPAZU076 | 773 | NDM | NDM-1 | - | MBL | PDC-16 | OXA-395 | - |
| CRPAZU077 | 233 | VIM | VIM-5 | - | MBL | PDC-3 | OXA-486 | OXA-4 |
| CRPAZU078 | 773 | NDM | NDM-1 | - | MBL | PDC-16 | OXA-395 | - |
| CRPAZU079 | 316 | VIM | VIM-2 | - | MBL | PDC-36 | OXA-395 | OXA-10/OXA-796 |
| CRPAZU080 | 654 | VIM | VIM-2 | - | MBL | PDC-3 | OXA-396 | - |
| CRPAZU081 | 1047 | IMP | IMP-1 | - | MBL | PDC-12 | OXA-488 | OXA-10 |
| CRPAZU082 | 4936 | IMP | IMP-1 | - | MBL | PDC-35 | OXA-50 | OXA-10/PAC-1 |
| CRPAZU083 | 1047 | IMP | IMP-1 |  | MBL | PDC-12 | OXA-488 | OXA-10 |
| CRPAZU084 | 235 | NDM | NDM-1 | PME-1 | MBL+ESBL | PDC-35 | OXA-488 | - |
| CRPAZU085 | 2167 | NEG | - | - | - | PDC-309 | OXA-1125 | - |
| CRPAZU086 | 244 | NDM | NDM-1 | PME-1 | MBL+ESBL | PDC-1 | OXA-847 | OXA-10 |
| CRPAZU087 | 244 | NDM | NDM-1 | PME-1 | MBL+ESBL | PDC-374 | OXA-847 | OXA-10 |
| CRPAZU088 | 235 | IMP | IMP-1 | - | MBL | PDC-35 | OXA-488 | - |
| CRPAZU089 | 664 | VIM | VIM-4 | - | MBL | PDC-98 | OXA-50 | - |
| CRPAZU090 | 941 | NEG | - | - | - | PDC-3 | OXA-396 | - |
| CRPAZU091 | 4936 | IMP | IMP-1 | - | MBL | PDC-35 | OXA-50 | OXA-10/PAC-1 |
| CRPAZU092 | 4936 | IMP | IMP-1 | - | MBL | PDC-35 | OXA-50 | OXA-10/PAC-1 |
| CRPAZU093 | 4936 | IMP | IMP-1 | - | MBL | PDC-35 | OXA-50 | OXA-10/PAC-1 |
| CRPAZU094 | 1047 | NEG | - | GES-13 | ESBL | PDC-12 | OXA-488 | OXA-10 |
| CRPAZU095 | 1047 | NEG | - | GES-13 | ESBL | PDC-12 | OXA-488 | OXA-10 |
| CRPAZU096 | 235 | IMP + NDM | NDM-1/IMP-1 | - | MBL | PDC-35 | OXA-488 | OXA-101 |
| CRPAZU097 | 4936 | IMP | IMP-1 | - | MBL | PDC-35 | OXA-50 | OXA-10/PAC-1 |
| CRPAZU098 | 235 | IMP + NDM | NDM-1/IMP-1 | - | MBL | PDC-35 | OXA-488 | OXA-101 |
| CRPAZU099 | 4936 | IMP | IMP-1 | - | MBL | PDC-35 | OXA-50 | OXA-10/PAC-1 |
| CRPAZU100 | 654 | NDM | NDM-1 |  | MBL | PDC-3 | OXA-396 | - |
| CRPAZU 101 | 395 | NEG | - | - | - | PDC-8 | OXA-905 | - |
| CRPAZU 102 | 235 | VIM | VIM-4 | - | MBL | PDC-35 | OXA-488 | OXA-35/CARB-2 |
| CRPAZU 103 | 317 | NEG | - | - | - | PDC-16 | OXA-488 | - |
| CRPAZU 104 | 5519 | NEG | - | - | - | PDC-35 | OXA-488 | - |
| CRPAZU 105 | 6220 | VIM | VIM-6 | - | MBL | PDC-1 | OXA-847 | OXA-10 |
| CRPAZU 106 | 6241 | NEG | - | PER-1 | ESBL | PDC-62 | OXA-395 | OXA-2 |
| CRPAZU 107 | 6678 | NEG | - | - | - | PDC-35 | OXA-488 | OXA-2 |
| CRPAZU 108 | 8141 | NEG | - | PER-1 | ESBL | PDC-33/62 | OXA-395 | OXA-2 |
| CRPAZU 109 | 8914 | NEG | - | - | - | PDC-3 | OXA-395 | OXA-10 |
| CRPAZU 110 | 23861 | KPC | KPC-2 | - | KPC | PDC-103 | OXA-50 | OXA-2/TEM-1A |
| CRPAZU 111 | 12914 | NEG | - | VEB-1 | ESBL | PDC-11 | OXA-846 | OXA-10 |
| CRPAZU 112 | 20176 | VIM | VIM-11 | - | MBL | PDC-36 | OXA-395 | OXA-2 |
| CRPAZU 113 | 20190 | NEG | - | - | - | PDC-240 | OXA-847 | - |
| CRPAZU 114 | 111 | VIM | VIM-2 | - | MBL | PDC-3 | OXA-395 | - |
| CRPAZU 115 | 111 | VIM | VIM-2 | - | MBL | PDC-3 | OXA-395 | - |
| CRPAZU 116 | 233 | VIM | VIM-2 | - | MBL | PDC-3 | OXA-486 | - |
| CRPAZU 117 | 235 | VIM | VIM-2 | - | MBL | PDC-35 | OXA-488 | - |
| CRPAZU 118 | 3511 | VIM | VIM-2 | - | MBL | PDC-3 | OXA-494 | - |
| CRPAZU 119 | 235 | NEG | GES-5 | - | GES-carba | PDC-35 | OXA-488 | - |
| CRPAZU 120 | 235 | VIM | VIM-1 | - | MBL | PDC-35 | OXA-488 | - |
| CRPAZU 121 | 654 | NDM | NDM-1/GES-5 | - | MBL+GES-carba | PDC-3 | OXA-396 | - |
| CRPAZU 122 | 773 | NDM | NDM-1 | - | MBL | PDC-16 | OXA-395 | rmtB4 |
| CRPAZU 123 | 773 | NDM | NDM-1 | - | MBL | PDC-16 | OXA-395 | rmtB4 |
| CRPAZU 124 | 155 | IMP | IMP-15 | - | MBL | PDC-374 | OXA-396 | OXA-10 |
| CRPAZU 125 | 277 | NEG | SPM-1 | - | MBL | PDC-374 | OXA-494 | rmtD1 |
| CRPAZU 126 | 1109 | NEG | - | - | - | PDC-3 | OXA-494 | - |
| CRPAZU 127 | 2351 | NEG | - | - | - | PDC-5 | OXA-50-like | - |
| CRPAZU 128 | 2351 | NEG | - | - | - | PDC-5 | OXA-50-like | - |
| CRPAZU 129 | 2351 | NEG | - | - | - | PDC-5 | OXA-50-like | - |
| CRPAZU 130 | 2351 | NEG | - | - | - | PDC-5 | OXA-50-like | - |
| CRPAZU 131 | 2351 | NEG | - | - | - | PDC-5 | OXA-50-like | - |
| CRPAZU 132 | 3321 | NEG | - | - | - | PDC-5 | OXA-914 | - |
| CRPAZU 133 | 1109 | NEG | - | - | - | PDC-3 | OXA-494 | - |
| CRPAZU 134 | 244 | NEG | - | - | - | PDC-5 | OXA-847 | - |
| CRPAZU 135 | 207 | NEG | - | - | - | PDC-30 | OXA-395 | - |
| CRPAZU 136 | 207 | NEG | - | - | - | PDC-30 | OXA-395 | - |

**Table S2. Disc diffusion growth inhibition zones (mm) of the carbapenem-resistant *P. aeruginosa* isolates**

| **Study number** | **Carbapenemase** | **ESBL** | **Group** | **TZP 30+6** | **TZP100+10** | **CAZ** | **FEP** | **ATM** | **IMP** | **MEM** | **CFD** | **C-T** | **C-A 10+4** | **C-A 40+10** | **C-E** | **I-R** | **M-V** | **CIP** | **LEV** | **TOB** | **AMI** | **GEN** |
| --- | --- | --- | --- | --- | --- | --- | --- | --- | --- | --- | --- | --- | --- | --- | --- | --- | --- | --- | --- | --- | --- | --- |
| CRPAZU0001 | - | - | - | 6 | 12 | 6 | 15 | 6 | 14 | 10 | 31 | 21 | 10 | 20 | 18 | 23 | 9 | 13 | 6 | 17 | 10 | 6 |
| CRPAZU002 | - | GES-12 | ESBL | 6 | 12 | 6 | 6 | 6 | 11 | 6 | 30 | 6 | 10 | 20 | 10 | 19 | 6 | 6 | 6 | 6 | 6 | 6 |
| CRPAZU003 | - | - | - | 6 | 17 | 6 | 10 | 6 | 6 | 9 | 32 | 30 | 13 | 33 | 15 | 20 | 19 | 22 | 15 | 14 | 6 | 6 |
| CRPAZU004 | VIM-4 | - | MBL | 6 | 9 | 6 | 6 | 23 | 6 | 6 | 31 | 6 | 8 | 17 | 6 | 6 | 6 | 6 | 6 | 6 | 6 | 6 |
| CRPAZU005 | - | - | - | 6 | 6 | 6 | 6 | 6 | 6 | 6 | 35 | 25 | 6 | 13 | 6 | 20 | 6 | 21 | 6 | 27 | 18 | 14 |
| CRPAZU006 | - | - | - | 6 | 6 | 6 | 11 | 6 | 8 | 6 | 28 | 19 | 8 | 10 | 6 | 19 | 6 | 11 | 6 | 17 | 9 | 14 |
| CRPAZU007 | IMP-18 | - | MBL | 24 | 26 | 6 | 14 | 31 | 19 | 28 | 27 | 8 | 7 | 11 | 6 | 17 | 24 | 34 | 27 | 16 | 26 | 11 |
| CRPAZU008 | VIM-4 | - | MBL | 13 | 13 | 6 | 9 | 22 | 7 | 6 | 32 | 6 | 12 | 13 | 6 | 8 | 6 | 20 | 12 | 6 | 6 | 6 |
| CRPAZU009 | IMP-7 | - | MBL | 11 | 19 | 6 | 6 | 13 | 6 | 6 | 21 | 6 | 6 | 6 | 6 | 9 | 6 | 7 | 7 | 7 | 16 | 6 |
| CRPAZU010 | IMP-1 | - | MBL | 6 | 12 | 6 | 6 | 16 | 6 | 6 | 23 | 6 | 6 | 6 | 6 | 6 | 6 | 6 | 6 | 6 | 6 | 6 |
| CRPAZU011 | - | - | - | 6 | 9 | 6 | 14 | 12 | 12 | 13 | 28 | 24 | 16 | 22 | 14 | 25 | 15 | 32 | 20 | 28 | 26 | 22 |
| CRPAZU012 | - | - | - | 6 | 6 | 6 | 7 | 6 | 7 | 10 | 33 | 23 | 9 | 24 | 14 | 25 | 13 | 44 | 40 | 26 | 18 | 16 |
| CRPAZU013 | VIM-2 | - | MBL | 13 | 22 | 6 | 18 | 22 | 6 | 6 | 30 | 6 | 11 | 17 | 17 | 6 | 8 | 9 | 6 | 6 | 14 | 19 |
| CRPAZU014 | - | - | - | 6 | 6 | 6 | 6 | 14 | 12 | 6 | 32 | 12 | 22 | 28 | 7 | 21 | 12 | 12 | 9 | 7 | 23 | 6 |
| CRPAZU015 | - | - | - | 6 | 6 | 6 | 9 | 15 | 11 | 10 | 28 | 22 | 21 | 28 | 12 | 21 | 13 | 9 | 8 | 10 | 23 | 6 |
| CRPAZU016 | - | - | - | 11 | 20 | 6 | 18 | 13 | 12 | 6 | 32 | 30 | 17 | 25 | 19 | 24 | 12 | 19 | 8 | 27 | 26 | 23 |
| CRPAZU017 | IMP-1 | - | MBL | 6 | 13 | 6 | 6 | 14 | 6 | 6 | 17 | 6 | 6 | 6 | 6 | 8 | 6 | 9 | 6 | 6 | 6 | 6 |
| CRPAZU018 | - | - | - | 6 | 10 | 6 | 15 | 13 | 11 | 14 | 30 | 23 | 19 | 25 | 6 | 25 | 18 | 12 | 10 | 10 | 27 | 13 |
| CRPAZU019 | OXA-181/IMP-26 | VEB-9 | MBL+OXA-48+ESBL | 6 | 9 | 6 | 6 | 6 | 6 | 6 | 26 | 6 | 6 | 6 | 6 | 6 | 6 | 6 | 6 | 13 | 6 | 6 |
| CRPAZU020 | - | - | - | 11 | 19 | 6 | 8 | 11 | 12 | 6 | 31 | 23 | 8 | 15 | 11 | 18 | 6 | 30 | 18 | 26 | 25 | 18 |
| CRPAZU021 | VIM-2 | - | MBL | 14 | 17 | 6 | 17 | 20 | 6 | 6 | 31 | 6 | 10 | 17 | 12 | 6 | 6 | 6 | 6 | 6 | 13 | 17 |
| CRPAZU022 | - | - | - | 6 | 11 | 10 | 20 | 15 | 9 | 13 | 28 | 26 | 18 | 23 | 15 | 25 | 17 | 34 | 27 | 26 | 22 | 17 |
| CRPAZU023 | - | - | - | 6 | 6 | 6 | 6 | 6 | 11 | 9 | 39 | 6 | 6 | 10 | 8 | 28 | 14 | 42 | 29 | 37 | 38 | 35 |
| CRPAZU024 | - | GES-12 | ESBL | 11 | 17 | 6 | 6 | 6 | 15 | 6 | 35 | 6 | 15 | 26 | 12 | 20 | 6 | 6 | 6 | 6 | 6 | 6 |
| CRPAZU025 | VIM-4 | - | MBL | 6 | 9 | 6 | 6 | 22 | 6 | 6 | 31 | 6 | 6 | 14 | 6 | 6 | 6 | 6 | 6 | 6 | 17 | 6 |
| CRPAZU026 | - | - | - | 18 | 22 | 6 | 6 | 6 | 12 | 20 | 41 | 17 | 19 | 30 | 20 | 29 | 28 | 18 | 11 | 22 | 14 | 12 |
| CRPAZU027 | - | - | - | 23 | 30 | 9 | 19 | 23 | 6 | 18 | 41 | 26 | 24 | 42 | 13 | 32 | 24 | 27 | 14 | 22 | 20 | 11 |
| CRPAZU028 | VIM-2 | - | MBL | 10 | 17 | 6 | 14 | 28 | 6 | 6 | 32 | 6 | 6 | 15 | 11 | 6 | 6 | 7 | 6 | 6 | 6 |  |
| CRPAZU029 | VIM-2 | - | MBL | 7 | 17 | 6 | 14 | 19 | 6 | 6 | 31 | 6 | 6 | 13 | 10 | 6 | 6 | 6 | 6 | 6 | 9 | 16 |
| CRPAZU030 | IMP-1 | - | MBL | 6 | 8 | 6 | 6 | 16 | 6 | 6 | 28 | 6 | 6 | 6 | 6 | 6 | 6 | 6 | 6 | 6 | 8 | 6 |
| CRPAZU031 | - | - | - | 6 | 6 | 6 | 8 | 10 | 10 | 6 | 27 | 15 | 6 | 6 | 13 | 20 | 9 | 8 | 6 | 12 | 26 | 16 |
| CRPAZU032 | VIM-1 | GES-7 | MBL+ESBL | 6 | 6 | 6 | 6 | 15 | 7 | 14 | 25 | 6 | 6 | 6 | 6 | 6 | 17 | 7 | 6 | 6 | 17 | 6 |
| CRPAZU033 | - | - | - | 6 | 13 | 6 | 6 | 6 | 12 | 6 | 35 | 6 | 9 | 18 | 16 | 22 | 7 | 6 | 6 | 6 | 6 | 6 |
| CRPAZU034 | - | - | - | 6 | 6 | 6 | 6 | 6 | 8 | 6 | 29 | 20 | 8 | 16 | 6 | 23 | 6 | 29 | 17 | 22 | 19 | 15 |
| CRPAZU035 | - | - | - | 18 | 26 | 13 | 13 | 6 | 8 | 6 | 34 | 27 | 20 | 24 | 24 | 25 | 13 | 32 | 19 | 28 | 24 | 20 |
| CRPAZU036 | VIM-2 | - | MBL | 10 | 17 | 6 | 14 | 20 | 6 | 6 | 32 | 6 | 6 | 11 | 11 | 6 | 6 | 6 | 6 | 6 | 6 | 17 |
| CRPAZU037 | - | - | - | 6 | 6 | 6 | 6 | 6 | 11 | 6 | 31 | 19 | 6 | 12 | 6 | 27 | 6 | 18 | 6 | 28 | 18 | 13 |
| CRPAZU038 | VIM-4 | - | MBL | 6 | 6 | 6 | 6 | 21 | 6 | 11 | 34 | 6 | 6 | 6 | 6 | 6 | 6 | 6 | 6 | 6 | 6 | 6 |
| CRPAZU039 | - | - | - | 6 | 11 | 6 | 6 | 6 | 12 | 7 | 32 | 27 | 11 | 19 | 8 | 28 | 15 | 22 | 7 | 30 | 29 | 25 |
| CRPAZU040 | - | - | - | 6 | 6 | 6 | 14 | 6 | 8 | 6 | 20 | 23 | 9 | 16 | 16 | 21 | 6 | 19 | 7 | 13 | 6 | 6 |
| CRPAZU041 | VIM-4 | - | MBL | 6 | 6 | 6 | 6 | 30 | 6 | 6 | 32 | 6 | 6 | 9 | 6 | 6 | 6 | 6 | 6 | 6 | 6 | 6 |
| CRPAZU042 | - | - | - | 6 | 6 | 6 | 6 | 6 | 6 | 9 | 31 | 25 | 11 | 25 | 6 | 22 | 15 | 10 | 6 | 6 | 6 | 6 |
| CRPAZU043 | - | - | - | 6 | 6 | 6 | 6 | 6 | 6 | 6 | 13 | 11 | 6 | 18 | 7 | 23 | 13 | 19 | 11 | 16 | 8 | 13 |
| CRPAZU044 | - | - | - | 20 | 28 | 6 | 6 | 23 | 6 | 9 | 36 | 35 | 27 | 37 | 7 | 25 | 19 | 22 | 12 | 19 | 18 | 13 |
| CRPAZU045 | - | VEB-16 | ESBL | 6 | 10 | 6 | 6 | 6 | 11 | 6 | 29 | 6 | 6 | 16 | 17 | 18 | 12 | 6 | 6 | 6 | 10 | 6 |
| CRPAZU046 | VIM-4 | - | MBL | 10 | 6 | 6 | 6 | 14 | 6 | 6 | 29 | 6 | 6 | 10 | 6 | 6 | 6 | 6 | 6 | 6 | 6 | 6 |
| CRPAZU047 | - | - | - | 6 | 14 | 6 | 6 | 6 | 9 | 6 | 39 | 30 | 19 | 29 | 11 | 23 | 6 | 17 | 9 | 17 | 11 | 6 |
| CRPAZU048 | - | VEB-9 | ESBL | 6 | 14 | 6 | 6 | 6 | 9 | 6 | 33 | 6 | 6 | 17 | 19 | 22 | 10 | 6 | 6 | 6 | 6 | 6 |
| CRPAZU049 | AIM-1 | SHV-5 | MBL+ESBL | 10 | 20 | 6 | 12 | 8 | 6 | 6 | 34 | 13 | 6 | 12 | 8 | 6 | 6 | 6 | 6 | 6 | 10 | 6 |
| CRPAZU050 | VIM-28 | SHV-2a | MBL+ESBL | 6 | 6 | 6 | 6 | 11 | 6 | 6 | 29 | 6 | 6 | 6 | 6 | 6 | 6 | 6 | 6 | 6 | 6 | 6 |
| CRPAZU051 | VIM-4 | - | MBL | 6 | 6 | 6 | 6 | 18 | 6 | 6 | 28 | 6 | 6 | 12 | 6 | 6 | 6 | 6 | 6 | 6 | 14 | 6 |
| CRPAZU052 | - | - | - | 6 | 6 | 6 | 17 | 6 | 13 | 11 | 34 | 27 | 15 | 23 | 12 | 28 | 14 | 6 | 6 | 17 | 20 | 17 |
| CRPAZU053 | VIM-2 | - | MBL | 6 | 12 | 6 | 6 | 14 | 6 | 6 | 28 | 6 | 6 | 6 | 6 | 10 | 6 | 6 | 6 | 6 | 6 | 6 |
| CRPAZU054 | VIM-4 | - | MBL | 6 | 6 | 6 | 6 | 16 | 6 | 6 | 30 | 6 | 6 | 8 | 6 | 6 | 6 | 6 | 6 | 6 | 6 | 6 |
| CRPAZU055 | VIM-4 | - | MBL | 6 | 6 | 6 | 6 | 15 | 6 | 6 | 29 | 6 | 6 | 6 | 6 | 6 | 6 | 6 | 6 | 6 | 6 | 6 |
| CRPAZU056 | VIM-1 | - | MBL | 6 | 15 | 6 | 6 | 33 | 6 | 6 | 25 | 6 | 6 | 6 | 6 | 8 | 6 | 6 | 6 | 10 | 17 | 15 |
| CRPAZU057 | - | - | - | 6 | 6 | 6 | 6 | 12 | 11 | 6 | 36 | 23 | 17 | 24 | 6 | 18 | 8 | 6 | 6 | 6 | 6 | 9 |
| CRPAZU058 | - | - | - | 18 | 23 | 8 | 16 | 13 | 13 | 6 | 30 | 31 | 17 | 20 | 18 | 27 | 6 | 31 | 18 | 26 | 26 | 20 |
| CRPAZU059 | - | VEB-9 | ESBL | 6 | 14 | 6 | 6 | 6 | 11 | 6 | 30 | 6 | 6 | 16 | 18 | 17 | 10 | 6 | 6 | 6 | 6 | 6 |
| CRPAZU060 | VIM-4 | - | MBL | 6 | 6 | 6 | 6 | 18 | 6 | 6 | 30 | 6 | 6 | 6 | 6 | 6 | 6 | 6 | 6 | 6 | 6 | 6 |
| CRPAZU061 | - | GES-52 | ESBL | 13 | 20 | 6 | 6 | 10 | 14 | 12 | 31 | 6 | 6 | 6 | 16 | 25 | 6 | 6 | 6 | 6 | 12 | 6 |
| CRPAZU062 | - | - | - | 6 | 6 | 6 | 10 | 6 | 13 | 9 | 30 | 21 | 10 | 19 | 10 | 29 | 12 | 38 | 26 | 31 | 29 | 28 |
| CRPAZU063 | VIM-2 | - | MBL | 6 | 12 | 6 | 10 | 24 | 6 | 6 | 28 | 6 | 11 | 20 | 13 | 6 | 6 | 6 | 6 | 6 | 14 | 6 |
| CRPAZU064 | - | - | - | 6 | 6 | 6 | 8 | 14 | 9 | 6 | 31 | 24 | 19 | 28 | 9 | 26 | 11 | 6 | 6 | 6 | 12 | 6 |
| CRPAZU065 | - | - | - | 6 | 14 | 13 | 14 | 23 | 10 | 15 | 34 | 25 | 24 | 29 | 14 | 23 | 18 | 6 | 6 | 6 | 14 | 6 |
| CRPAZU066 | VIM-2 | - | MBL | 6 | 17 | 6 | 10 | 17 | 6 | 6 | 28 | 6 | 6 | 12 | 13 | 6 | 6 | 6 | 6 | 6 | 6 | 17 |
| CRPAZU067 | NDM-1/VIM-2 | - | MBL | 6 | 6 | 6 | 6 | 20 | 6 | 6 | 26 | 6 | 6 | 6 | 6 | 6 | 6 | 6 | 6 | 6 | 6 | 6 |
| CRPAZU068 | IMP-1 | - | MBL | 6 | 15 | 6 | 6 | 30 | 6 | 6 | 31 | 6 | 6 | 6 | 6 | 6 | 6 | 6 | 6 | 6 | 6 | 6 |
| CRPAZU069 | - | - | - | 6 | 6 | 6 | 7 | 6 | 10 | 6 | 31 | 23 | 6 | 16 | 11 | 26 | 6 | 15 | 6 | 29 | 25 | 23 |
| CRPAZU070 | - | - | - | 6 | 13 | 6 | 15 | 12 | 12 | 17 | 31 | 29 | 19 | 30 | 14 | 29 | 20 | 37 | 36 | 25 | 24 | 25 |
| CRPAZU071 | VIM-5 | VEB-14 | MBL+ESBL | 6 | 8 | 6 | 6 | 6 | 6 | 6 | 22 | 6 | 6 | 6 | 9 | 6 | 6 | 8 | 6 | 6 | 6 | 6 |
| CRPAZU072 | IMP-1 | - | MBL | 6 | 10 | 6 | 6 | 18 | 16 | 6 | 28 | 6 | 6 | 6 | 6 | 18 | 6 | 6 | 6 | 6 | 6 | 6 |
| CRPAZU073 | VIM-4 | - | MBL | 6 | 6 | 6 | 6 | 31 | 6 | 6 | 31 | 6 | 6 | 6 | 6 | 6 | 6 | 6 | 6 | 6 | 6 | 6 |
| CRPAZU074 | VIM-2 | - | MBL | 6 | 15 | 6 | 6 | 19 | 6 | 6 | 24 | 6 | 6 | 9 | 7 | 6 | 6 | 6 | 6 | 6 | 6 | 6 |
| CRPAZU075 | - | PER-1 | ESBL | 6 | 6 | 6 | 6 | 6 | 6 | 6 | 26 | 6 | 6 | 11 | 6 | 20 | 7 | 6 | 6 | 6 | 8 | 11 |
| CRPAZU076 | NDM-1 | - | MBL | 6 | 7 | 6 | 6 | 19 | 6 | 6 | 23 | 6 | 6 | 6 | 6 | 6 | 6 | 6 | 6 | 6 | 6 | 6 |
| CRPAZU077 | VIM-5 | - | MBL | 6 | 6 | 6 | 6 | 25 | 6 | 6 | 27 | 6 | 6 | 6 | 6 | 6 | 6 | 6 | 6 | 6 | 6 | 6 |
| CRPAZU078 | NDM-1 | - | MBL | 6 | 6 | 6 | 6 | 34 | 6 | 6 | 21 | 6 | 6 | 6 | 6 | 6 | 6 | 6 | 6 | 6 | 6 | 6 |
| CRPAZU079 | VIM-2 | - | MBL | 6 | 6 | 6 | 6 | 16 | 6 | 6 | 26 | 6 | 6 | 14 | 11 | 6 | 6 | 6 | 6 | 6 | 6 | 6 |
| CRPAZU080 | VIM-2 | - | MBL | 8 | 14 | 6 | 8 | 17 | 6 | 6 | 26 | 6 | 6 | 6 | 6 | 6 | 6 | 6 | 6 | 6 | 22 | 6 |
| CRPAZU081 | IMP-1 | - | MBL | 9 | 16 | 6 | 6 | 19 | 6 | 6 | 28 | 6 | 6 | 6 | 6 | 6 | 6 | 6 | 7 | 6 | 12 | 6 |
| CRPAZU082 | IMP-1 | - | MBL | 6 | 12 | 6 | 6 | 15 | 6 | 6 | 26 | 6 | 6 | 6 | 6 | 6 | 6 | 6 | 6 | 6 | 6 | 6 |
| CRPAZU083 | IMP-1 |  | MBL | 6 | 11 | 6 | 6 | 19 | 6 | 6 | 26 | 6 | 6 | 6 | 6 | 6 | 6 | 6 | 6 | 6 | 8 | 8 |
| CRPAZU084 | NDM-1 | PME-1 | MBL+ESBL | 6 | 9 | 6 | 6 | 6 | 14 | 6 | 25 | 6 | 6 | 6 | 6 | 19 | 6 | 6 | 6 | 6 | 6 | 6 |
| CRPAZU085 | - | - | - | 6 | 13 | 6 | 6 | 6 | 12 | 6 | 16 | 6 | 6 | 6 | 8 | 26 | 6 | 28 | 18 | 24 | 17 | 12 |
| CRPAZU086 | NDM-1 | PME-1 | MBL+ESBL | 6 | 11 | 6 | 6 | 6 | 6 | 6 | 25 | 6 | 6 | 6 | 6 | 6 | 6 | 6 | 6 | 25 | 6 | 6 |
| CRPAZU087 | NDM-1 | PME-1 | MBL+ESBL | 6 | 9 | 6 | 6 | 6 | 6 | 6 | 17 | 6 | 6 | 6 | 6 | 6 | 6 | 6 | 6 | 24 | 6 | 6 |
| CRPAZU088 | IMP-1 | - | MBL | 12 | 18 | 6 | 6 | 18 | 18 | 6 | 26 | 6 | 6 | 6 | 6 | 23 | 6 | 6 | 6 | 6 | 18 | 6 |
| CRPAZU089 | VIM-4 | - | MBL | 6 | 12 | 6 | 10 | 19 | 6 | 6 | 25 | 6 | 6 | 12 | 8 | 6 | 6 | 6 | 6 | 14 | 15 | 6 |
| CRPAZU090 | - | - | - | 6 | 20 | 6 | 11 | 6 | 6 | 6 | 36 | 31 | 19 | 23 | 9 | 22 | 6 | 20 | 10 | 30 | 25 | 23 |
| CRPAZU091 | IMP-1 | - | MBL | 6 | 10 | 6 | 6 | 14 | 6 | 6 | 22 | 6 | 6 | 6 | 6 | 6 | 6 | 6 | 6 | 6 | 6 | 6 |
| CRPAZU092 | IMP-1 | - | MBL | 6 | 10 | 6 | 6 | 11 | 6 | 6 | 22 | 6 | 6 | 6 | 6 | 8 | 6 | 6 | 6 | 6 | 6 | 6 |
| CRPAZU093 | IMP-1 | - | MBL | 6 | 10 | 6 | 6 | 14 | 6 | 6 | 19 | 6 | 6 | 6 | 6 | 6 | 6 | 6 | 6 | 6 | 6 | 6 |
| CRPAZU094 | - | GES-13 | ESBL | 6 | 6 | 6 | 7 | 6 | 10 | 6 | 26 | 19 | 16 | 24 | 15 | 21 | 6 | 6 | 6 | 6 | 6 | 6 |
| CRPAZU095 | - | GES-13 | ESBL | 6 | 6 | 6 | 8 | 7 | 7 | 6 | 26 | 20 | 16 | 25 | 17 | 20 | 6 | 6 | 6 | 6 | 8 | 6 |
| CRPAZU096 | NDM-1/IMP-1 | - | MBL | 6 | 14 | 6 | 6 | 19 | 6 | 6 | 24 | 6 | 6 | 6 | 6 | 6 | 6 | 6 | 6 | 9 | 21 | 6 |
| CRPAZU097 | IMP-1 | - | MBL | 12 | 20 | 6 | 6 | 17 | 6 | 6 | 17 | 6 | 6 | 6 | 6 | 6 | 6 | 6 | 6 | 10 | 20 | 6 |
| CRPAZU098 | NDM-1/IMP-1 | - | MBL | 6 | 13 | 6 | 6 | 18 | 6 | 6 | 26 | 6 | 6 | 6 | 6 | 6 | 6 | 11 | 6 | 11 | 19 | 6 |
| CRPAZU099 | IMP-1 | - | MBL | 6 | 9 | 6 | 6 | 14 | 6 | 6 | 23 | 6 | 6 | 6 | 6 | 6 | 6 | 6 | 6 | 6 | 6 | 6 |
| CRPAZU100 | NDM-1 | - | MBL | 6 | 10 | 6 | 6 | 26 | 6 | 6 | 23 | 6 | 6 | 6 | 6 | 6 | 6 | 6 | 6 | 6 | 6 | 21 |
| CRPAZU 101 | - | - | - | 6 | 18 | 6 | 12 | 9 | 10 | 6 | 31 | 28 | 14 | 15 | 19 | 13 | 6 | 29 | 18 | 24 | 16 | 16 |
| CRPAZU 102 | VIM-4 | - | MBL | 6 | 6 | 6 | 6 | 7 | 6 | 6 | 27 | 6 | 6 | 6 | 6 | 6 | 6 | 6 | 6 | 8 | 9 | 7 |
| CRPAZU 103 | - | - | - | 6 | 6 | 6 | 11 | 10 | 9 | 16 | 27 | 26 | 14 | 26 | 18 | 28 | 19 | 13 | 8 | 30 | 27 | 24 |
| CRPAZU 104 | - | - | - | 6 | 12 | 6 | 19 | 18 | 10 | 7 | 33 | 30 | 22 | 24 | 24 | 25 | 9 | 6 | 6 | 6 | 6 | 6 |
| CRPAZU 105 | VIM-6 | - | MBL | 6 | 6 | 6 | 6 | 6 | 6 | 6 | 24 | 6 | 6 | 6 | 6 | 6 | 6 | 6 | 6 | 6 | 6 | 6 |
| CRPAZU 106 | - | PER-1 | ESBL | 6 | 21 | 6 | 6 | 6 | 10 | 13 | 32 | 14 | 16 | 26 | 20 | 23 | 10 | 20 | 13 | 7 | 15 | 6 |
| CRPAZU 107 | - | - | - | 6 | 8 | 6 | 15 | 15 | 11 | 6 | 28 | 25 | 14 | 28 | 24 | 27 | 7 | 9 | 6 | 9 | 21 | 6 |
| CRPAZU 108 | - | PER-1 | ESBL | 6 | 22 | 6 | 6 | 6 | 8 | 12 | 35 | 15 | 14 | 23 | 21 | 27 | 13 | 18 | 6 | 6 | 11 | 6 |
| CRPAZU 109 | - | - | - | 6 | 15 | 6 | 15 | 17 | 11 | 12 | 32 | 31 | 26 | 31 | 22 | 23 | 17 | 12 | 6 | 6 | 13 | 6 |
| CRPAZU 110 | KPC-2 | - | KPC | 10 | 14 | 6 | 6 | 6 | 6 | 6 | 30 | 26 | 25 | 28 | 13 | 22 | 6 | 6 | 6 | 18 | 32 | 17 |
| CRPAZU 111 | - | VEB-1 | ESBL | 6 | 16 | 6 | 6 | 6 | 15 | 6 | 30 | 6 | 6 | 12 | 24 | 27 | 11 | 6 | 6 | 6 | 10 | 6 |
| CRPAZU 112 | VIM-11 | - | MBL | 6 | 12 | 6 | 6 | 20 | 9 | 24 | 26 | 6 | 6 | 6 | 16 | 14 | 26 | 6 | 6 | 6 | 9 | 6 |
| CRPAZU 113 | - | - | - | 7 | 6 | 6 | 6 | 6 | 13 | 6 | 11 | 12 | 6 | 6 | 6 | 24 | 12 | 17 | 11 | 11 | 6 | 6 |
| CRPAZU 114 | VIM-2 | - | MBL | 6 | 13 | 6 | 10 | 17 | 6 | 6 | 40 | 6 | 6 | 14 | 13 | 6 | 6 | 6 | 6 | 6 | 6 | 13 |
| CRPAZU 115 | VIM-2 | - | MBL | 8 | 16 | 6 | 10 | 24 | 6 | 16 | 32 | 6 | 6 | 11 | 16 | 10 | 15 | 6 | 6 | 6 | 6 | 13 |
| CRPAZU 116 | VIM-2 | - | MBL | 6 | 6 | 6 | 6 | 23 | 6 | 11 | 30 | 6 | 6 | 6 | 12 | 6 | 11 | 6 | 6 | 6 | 6 | 6 |
| CRPAZU 117 | VIM-2 | - | MBL | 7 | 17 | 6 | 18 | 26 | 6 | 6 | 33 | 6 | 13 | 21 | 25 | 6 | 6 | 13 | 8 | 19 | 6 | 24 |
| CRPAZU 118 | VIM-2 | - | MBL | 6 | 10 | 6 | 17 | 12 | 6 | 6 | 27 | 6 | 7 | 20 | 19 | 6 | 6 | 11 | 6 | 7 | 6 | 6 |
| CRPAZU 119 | GES-5 | - | GES-carba | 6 | 14 | 6 | 17 | 14 | 6 | 6 | 32 | 23 | 23 | 29 | 24 | 17 | 6 | 6 | 6 | 6 | 10 | 6 |
| CRPAZU 120 | VIM-1 | - | MBL | 6 | 10 | 6 | 6 | 25 | 6 | 6 | 29 | 6 | 6 | 6 | 6 | 6 | 6 | 20 | 8 | 8 | 17 | 8 |
| CRPAZU 121 | NDM-1/GES-5 | - | MBL+GES-carba | 6 | 11 | 6 | 6 | 19 | 6 | 6 | 24 | 6 | 6 | 6 | 6 | 6 | 6 | 6 | 6 | 23 | 6 | 19 |
| CRPAZU 122 | NDM-1 | - | MBL | 6 | 6 | 6 | 6 | 37 | 6 | 6 | 30 | 6 | 6 | 7 | 6 | 6 | 6 | 6 | 6 | 6 | 6 | 6 |
| CRPAZU 123 | NDM-1 | - | MBL | 6 | 6 | 6 | 6 | 30 | 6 | 6 | 23 | 6 | 6 | 6 | 6 | 6 | 6 | 6 | 6 | 6 | 7 | 6 |
| CRPAZU 124 | IMP-15 | - | MBL | 10 | 19 | 6 | 6 | 26 | 21 | 16 | 29 | 6 | 6 | 6 | 8 | 23 | 19 | 23 | 17 | 17 | 26 | 13 |
| CRPAZU 125 | SPM-1 | - | MBL | 6 | 10 | 6 | 6 | 20 | 6 | 6 | 19 | 6 | 6 | 6 | 6 | 6 | 6 | 6 | 6 | 6 | 6 | 6 |
| CRPAZU 126 | - | - | - | 6 | 8 | 6 | 19 | 13 | 11 | 15 | 31 | 29 | 19 | 29 | 22 | 26 | 15 | 32 | 28 | 27 | 24 | 20 |
| CRPAZU 127 | - | - | - | 6 | 6 | 6 | 6 | 6 | 9 | 6 | 24 | 26 | 6 | 18 | 13 | 22 | 6 | 27 | 12 | 23 | 20 | 18 |
| CRPAZU 128 | - | - | - | 6 | 12 | 6 | 15 | 13 | 12 | 9 | 28 | 28 | 14 | 24 | 19 | 25 | 8 | 28 | 14 | 25 | 26 | 23 |
| CRPAZU 129 | - | - | - | 6 | 6 | 6 | 12 | 8 | 10 | 6 | 30 | 32 | 15 | 16 | 27 | 23 | 6 | 31 | 16 | 22 | 23 | 21 |
| CRPAZU 130 | - | - | - | 6 | 8 | 6 | 12 | 9 | 10 | 6 | 26 | 26 | 13 | 17 | 16 | 22 | 6 | 28 | 15 | 21 | 23 | 21 |
| CRPAZU 131 | - | - | - | 6 | 13 | 6 | 13 | 9 | 9 | 6 | 26 | 28 | 14 | 19 | 19 | 25 | 6 | 31 | 18 | 26 | 24 | 24 |
| CRPAZU 132 | - | - | - | 7 | 16 | 6 | 18 | 18 | 12 | 21 | 35 | 31 | 21 | 31 | 28 | 29 | 22 | 38 | 37 | 31 | 30 | 27 |
| CRPAZU 133 | - | - | - | 6 | 14 | 6 | 17 | 13 | 9 | 16 | 28 | 29 | 19 | 28 | 23 | 28 | 18 | 36 | 31 | 27 | 23 | 23 |
| CRPAZU 134 | - | - | - | 6 | 6 | 6 | 18 | 15 | 12 | 19 | 32 | 31 | 24 | 30 | 26 | 32 | 20 | 40 | 40 | 29 | 27 | 30 |
| CRPAZU 135 | - | - | - | 6 | 8 | 6 | 14 | 6 | 12 | 6 | 30 | 28 | 6 | 21 | 17 | 29 | 7 | 31 | 23 | 29 | 22 | 23 |
| CRPAZU 136 | - | - | - | 6 | 14 | 6 | 20 | 14 | 12 | 6 | 31 | 30 | 16 | 25 | 22 | 22 | 6 | 28 | 18 | 26 | 20 | 20 |

TZP, Piperacillin-Tazobactam; CAZ, Ceftazidime; FEP, Cefepime; ATM, Aztreonam; IMP, Imipenem; MEM, Meropenem; C-T, Ceftolozane-Tazobactam; C-A, Ceftazidime-Avibactam, I-R, Imipenem-Relebactam; C-E, Cefepime-Enmetazobactam; M-V, Meropenem-Vaborbactam; CIP, Ciprofloxacin; LEV, Levofloxacin; TOB, Tobramycin; AMI, Amikacin; GEN, Gentamicin.

**Table S3. Double disc synergy testing of the carbapenem-resistant *P. aeruginosa* isolates**

| **Study Number** | **Group** | **Carbapenemase** | **ESBL** | **PDC** | **OXA** | **Others** | **C-T** | **I-R** | **C-E** | **FEP** | **Synergy C-E/FEP 12 mm** | **Synergy I-R/FEP 12 mm** |
| --- | --- | --- | --- | --- | --- | --- | --- | --- | --- | --- | --- | --- |
| CRPAZU001 | - | - | - | PDC-33 | OXA-488 | - | 21 | 23 | 18 | 15 | - | - |
| CRPAZU002 | ESBL | - | GES-12 | PDC-98 | OXA-50 | - | 6 | 19 | 10 | 6 | - | +++ |
| CRPAZU006 | - | - | - | PDC-6 | OXA-50 | - | 19 | 19 | 6 | 11 | - | - |
| CRPAZU007 | MBL | IMP-18 | - | PDC-5 | OXA-494 | OXA-10 | 8 | 17 | 6 | 14 | - | ++ |
| CRPAZU014 | - | - | - | PDC-36 | OXA-395 | OXA-10 | 12 | 21 | 7 | 6 | - | - |
| CRPAZU015 | - | - | - | PDC-36 | OXA-395 | OXA-10 | 22 | 21 | 12 | 9 | - | - |
| CRPAZU023 | - | - | - | PDC-374 | OXA-396 | - | 6 | 28 | 8 | 6 | - | - |
| CRPAZU024 | ESBL | - | GES-12 | PDC98 | OXA-50 | - | 6 | 20 | 12 | 6 | - | +++ |
| CRPAZU027 | - | - | - | PDC-374 | OXA-905 | - | 17 | 29 | 20 | 6 | - | + |
| CRPAZU031 | - | - | - | PDC-3 | OXA-395 | OXA-14/CARB-2 | 15 | 20 | 13 | 8 | - | + |
| CRPAZU033 | - | - | - | PDC-23 | OXA-904 | - | 6 | 22 | 16 | 6 | - | - |
| CRPAZU034 | - | - | - | PDC-24 | OXA-486 | - | 20 | 23 | 6 | 6 | - | - |
| CRPAZU037 | - | - | - | PDC-374 | OXA-486 | - | 19 | 27 | 6 | 6 | - | - |
| CRPAZU043 | - | - | - | PDC97 | OXA-50 | - | 11 | 23 | 7 | 6 | - | - |
| CRPAZU045 | ESBL | - | VEB-16 | PDC-35 | OXA-488 | OXA-10 | 6 | 18 | 17 | 6 | ++ | + |
| CRPAZU048 | ESBL | - | VEB-9 | PDC11 | OXA-846 | OXA-10 | 6 | 22 | 19 | 6 | ++ | + |
| CRPAZU059 | ESBL | - | VEB-9 | PDC-11 | OXA-846 | OXA-10 | 6 | 17 | 18 | 6 | ++ | + |
| CRPAZU061 | ESBL | - | GES-52 | PDC-35 | OXA-488 | - | 6 | 25 | 16 | 6 | - | +++ |
| CRPAZU062 | - | - | - | PDC-8 | OXA-395 | - | 21 | 29 | 10 | 10 | - | + |
| CRPAZU072 | MBL | IMP-1 | - | PDC-12 | OXA-488 | OXA-10 | 6 | 18 | 6 | 6 | - | - |
| CRPAZU075 | ESBL | - | PER-1 | PDC-8 | OXA-396 | OXA-4 | 6 | 20 | 6 | 6 | - | - |
| CRPAZU084 | MBL+ESBL | NDM-1 | PME-1 | PDC-35 | OXA-488 | - | 6 | 19 | 6 | 6 | - | - |
| CRPAZU085 | - | - | - | PDC-309 | OXA-486 | - | 6 | 26 | 8 | 6 | - | - |
| CRPAZU088 | MBL | IMP-1 | - | PDC-35 | OXA-488 | - | 6 | 23 | 6 | 6 | - | - |
| CRPAZU094 | ESBL | - | GES-13 | PDC-12 | OXA-488 | OXA-10 | 19 | 21 | 21 | 7 | - | + |
| CRPAZU095 | ESBL | - | GES-13 | PDC-12 | OXA-488 | OXA-10 | 20 | 20 | 17 | 8 | + | ++ |
| CRPAZU106 | ESBL | - | PER-1 | PDC-62 | OXA-395 | OXA-2 | 14 | 23 | 16 | 6 | ++ | + |
| CRPAZU108 | ESBL | - | PER-1 | PDC-33/62 | OXA-395 | OXA-2 | 15 | 27 | 15 | 6 | - | - |
| CRPAZU111 | ESBL | - | VEB-1 | PDC-11 | OXA-846 | OXA-10 | 6 | 27 | 24 | 6 | +++ | +++ |
| CRPAZU113 | - | - | - | PDC-240 | OXA-847 | - | 12 | 24 | 6 | 6 | - | - |
| CRPAZU124 | MBL | IMP-15 | - | PDC-374 | OXA-396 | OXA-10 | 6 | 23 | 8 | 6 | - | - |

C-T, Ceftolozane-Tazobactam; I-R, Imipenem-Relebactam; C-E, Cefepime-Enmetazobactam; FEP, Cefepime

ESBL-producers are highlighted in yellow. A ‘plus’ symbol indicates the degree of the halo detected between the C-E, I-R and FEP discs, while a ‘minus’ refers to no detection. Results that match expected outcomes are marked in green, while discrepancies are shown in red.

**Table S4. MICs (mg/L) of the CRPA isolates as determined by strip gradient test.**

| **Study number** | **Carbapenemase** | **ESBL** | **Group** | **C-E** | **C-T** | **M-V** | **C-A** | **I-R** | **A-A** |
| --- | --- | --- | --- | --- | --- | --- | --- | --- | --- |
| CRPAZU0001 | - | - | - | >64 | 6 | 8 | 16 | 1,5 | >256 |
| CRPAZU002 | - | GES-12 | ESBL | >64 | >256 | 96 | 48 | 8 | 24 |
| CRPAZU003 | - | - | - | >64 | 2 | 4 | 1,5 | 1,5 | >256 |
| CRPAZU004 | VIM-4 | - | MBL | >64 | >256 | >256 | >256 | >32 | 6 |
| CRPAZU005 | - | - | - | >64 | 6 | 48 | >256 | 6 | 24 |
| CRPAZU006 | - | - | - | >64 | 8 | >256 | >256 | 8 | 16 |
| CRPAZU007 | IMP-18 | - | MBL | >64 | >256 | 1 | >256 | 3 | 1.5 |
| CRPAZU008 | VIM-4 | - | MBL | >64 | >256 | >256 | 64 | >32 | 12 |
| CRPAZU009 | IMP-7 | - | MBL | >64 | >256 | >256 | >256 | >32 | 48 |
| CRPAZU010 | IMP-1 | - | MBL | >64 | >256 | >256 | >256 | >32 | 32 |
| CRPAZU011 | - | - | - | >64 | 4 | 24 | 8 | 1,5 | 24 |
| CRPAZU012 | - | - | - | >64 | 8 | 12 | 12 | 1,5 | 32 |
| CRPAZU013 | VIM-2 | - | MBL | 48 | >256 | 96 | >256 | >32 | 12 |
| CRPAZU014 | - | - | - | >64 | 24 | >256 | 4 | 12 | >256 |
| CRPAZU015 | - | - | - | >64 | 12 | 48 | 3 | 6 | 64 |
| CRPAZU016 | - | - | - | >64 | 2 | 48 | 8 | 6 | 64 |
| CRPAZU017 | IMP-1 | - | MBL | >64 | >256 | >256 | >256 | >32 | 64 |
| CRPAZU018 | - | - | - | >64 | 4 | 16 | 4 | 7 | 12 |
| CRPAZU019 | OXA-181/IMP-26 | VEB-9 | MBL+OXA-48+ESBL | >64 | >256 | >256 | >256 | >32 | >256 |
| CRPAZU020 | - | - | - | >64 | 8 | 32 | 96 | 12 | >256 |
| CRPAZU021 | VIM-2 | - | MBL | >64 | >256 | >256 | 64 | >32 | 16 |
| CRPAZU022 | - | - | - | 48 | 1,5 | 12 | 4 | 1,5 | 16 |
| CRPAZU023 | - | - | - | >64 | >256 | 24 | >256 | 1,5 | >256 |
| CRPAZU024 | - | GES-12 | ESBL | >64 | >256 | 32 | 6 | 3 | 8 |
| CRPAZU025 | VIM-4 | - | MBL | >64 | >256 | >256 | >256 | >32 | 8 |
| CRPAZU026 | - | - | - | >64 | 1,5 | 3 | 3 | 1,5 | 32 |
| CRPAZU027 | - | - | - | >64 | 2 | 8 | 2 | 2 | 0.5 |
| CRPAZU028 | VIM-2 | - | MBL | >64 | >256 | 96 | >256 | >32 | 4 |
| CRPAZU029 | VIM-2 | - | MBL | >64 | >256 | >256 | 64 | >32 | 16 |
| CRPAZU030 | IMP-1 | - | MBL | >64 | >256 | >256 | >256 | >32 | 24 |
| CRPAZU031 | - | - | - | >64 | 48 | 24 | >256 | 3 | 32 |
| CRPAZU032 | VIM-1 | GES-7 | MBL+ESBL | >64 | >256 | 6 | >256 | >32 | 3 |
| CRPAZU033 | - | - | - | >64 | >256 | 24 | 8 | 3 | >256 |
| CRPAZU034 | - | - | - | >64 | 8 | 96 | 48 | 3 | >256 |
| CRPAZU035 | - | - | - | >64 | 2 | 8 | 3 | 2 | >256 |
| CRPAZU036 | VIM-2 | - | MBL | >64 | >256 | >256 | 96 | >32 | 16 |
| CRPAZU037 | - | - | - | >64 | 48 | 64 | >256 | 3 | >256 |
| CRPAZU038 | VIM-4 | - | MBL | >64 | >256 | >256 | >256 | >32 | 12 |
| CRPAZU039 | - | - | - | >64 | 2 | 12 | 16 | 0,75 | >256 |
| CRPAZU040 | - | - | - | >64 | 16 | 96 | >256 | 4 | >256 |
| CRPAZU041 | VIM-4 | - | MBL | >64 | >256 | >256 | >256 | >32 | 4 |
| CRPAZU042 | - | - | - | >64 | 1 | 48 | 6 | 1,5 | 0.5 |
| CRPAZU043 | - | - | - | >64 | 16 | 12 | 24 | 3 | >256 |
| CRPAZU044 | - | - | - | >64 | 0,75 | 1,5 | 1 | 0,75 | 4 |
| CRPAZU045 | - | VEB-16 | ESBL | >64 | >256 | >256 | >256 | 3 | >256 |
| CRPAZU046 | VIM-4 | - | MBL | >64 | >256 | >256 | >256 | >32 | 8 |
| CRPAZU047 | - | - | - | >64 | 2 | >256 | 2 | 2 | >256 |
| CRPAZU048 | - | VEB-9 | ESBL | >64 | >256 | 24 | 64 | 2 | 128 |
| CRPAZU049 | AIM-1 | SHV-5 | MBL+ESBL | >64 | >256 | >256 | >256 | >32 | 16 |
| CRPAZU050 | VIM-28 | SHV-2a | MBL+ESBL | >64 | >256 | >256 | >256 | >32 | 16 |
| CRPAZU051 | VIM-4 | - | MBL | >64 | >256 | >256 | >256 | >32 | 12 |
| CRPAZU052 | - | - | - | >64 | 8 | 16 | 12 | 1 | 32 |
| CRPAZU053 | VIM-2 | - | MBL | >64 | >256 | 64 | >256 | >32 | 32 |
| CRPAZU054 | VIM-4 | - | MBL | >64 | >256 | >256 | >256 | >32 | 16 |
| CRPAZU055 | VIM-4 | - | MBL | >64 | >256 | >256 | >256 | >32 | 8 |
| CRPAZU056 | VIM-1 | - | MBL | >64 | >256 | 48 | >256 | >32 | 3 |
| CRPAZU057 | - | - | - | >64 | 12 | 48 | 12 | 6 | 64 |
| CRPAZU058 | - | - | - | >64 | 1,5 | 64 | 12 | 1,5 | >256 |
| CRPAZU059 | - | VEB-9 | ESBL | >64 | >256 | 96 | >256 | 2 | >256 |
| CRPAZU060 | VIM-4 | - | MBL | >64 | >256 | >256 | >256 | >32 | 12 |
| CRPAZU061 | - | GES-52 | ESBL | >64 | >256 | 16 | >256 | >32 | 96 |
| CRPAZU062 | - | - | - | >64 | 8 | 48 | 12 | 1 | >256 |
| CRPAZU063 | VIM-2 | - | MBL | >64 | >256 | 128 | 24 | >32 | 4 |
| CRPAZU064 | - | - | - | >64 | 16 | 32 | 2 | 3 | 24 |
| CRPAZU065 | - | - | - | >64 | 4 | 8 | 1,5 | 2 | 6 |
| CRPAZU066 | VIM-2 | - | MBL | >64 | >256 | >256 | 48 | >32 | 12 |
| CRPAZU067 | NDM-1/VIM-2 | - | MBL | >64 | >256 | >256 | >256 | >32 | 6 |
| CRPAZU068 | IMP-1 | - | MBL | >64 | >256 | >256 | >256 | >32 | 8 |
| CRPAZU069 | - | - | - | >64 | 3 | >256 | 96 | 2 | >256 |
| CRPAZU070 | - | - | - | 24 | 2 | 6 | 3 | 1 | 6 |
| CRPAZU071 | VIM-5 | VEB-14 | MBL+ESBL | >64 | >256 | >256 | >256 | >32 | >256 |
| CRPAZU072 | IMP-1 | - | MBL | >64 | >256 | 192 | >256 | >32 | 16 |
| CRPAZU073 | VIM-4 | - | MBL | >64 | >256 | >256 | >256 | >32 | 4 |
| CRPAZU074 | VIM-2 | - | MBL | >64 | >256 | >256 | >256 | >32 | 16 |
| CRPAZU075 | - | PER-1 | ESBL | >64 | >256 | >256 | >256 | >32 | >256 |
| CRPAZU076 | NDM-1 | - | MBL | >64 | >256 | >256 | >256 | >32 | 12 |
| CRPAZU077 | VIM-5 | - | MBL | >64 | >256 | >256 | >256 | >32 | 6 |
| CRPAZU078 | NDM-1 | - | MBL | >64 | >256 | >256 | >256 | >32 | 4 |
| CRPAZU079 | VIM-2 | - | MBL | >64 | >256 | >256 | 32 | >32 | 32 |
| CRPAZU080 | VIM-2 | - | MBL | >64 | >256 | >256 | >256 | >32 | 24 |
| CRPAZU081 | IMP-1 | - | MBL | >64 | >256 | >256 | >256 | >32 | 24 |
| CRPAZU082 | IMP-1 | - | MBL | >64 | >256 | >256 | >256 | >32 | 3 |
| CRPAZU083 | IMP-1 |  | MBL | >64 | >256 | >256 | >256 | >32 | 16 |
| CRPAZU084 | NDM-1 | PME-1 | MBL+ESBL | >64 | >256 | 256 | >256 | >32 | >256 |
| CRPAZU085 | - | - | - | >64 | >256 | >256 | >256 | 4 | >256 |
| CRPAZU086 | NDM-1 | PME-1 | MBL+ESBL | >64 | >256 | >256 | >256 | >32 | 64 |
| CRPAZU087 | NDM-1 | PME-1 | MBL+ESBL | >64 | >256 | >256 | >256 | >32 | >256 |
| CRPAZU088 | IMP-1 | - | MBL | >64 | >256 | 64 | >256 | 3 | 16 |
| CRPAZU089 | VIM-4 | - | MBL | >64 | >256 | 256 | >256 | >32 | 16 |
| CRPAZU090 | - | - | - | >64 | 2 | 32 | 8 | 4 | >256 |
| CRPAZU091 | IMP-1 | - | MBL | >64 | >256 | >256 | >256 | >32 | 32 |
| CRPAZU092 | IMP-1 | - | MBL | >64 | >256 | >256 | >256 | >32 | 32 |
| CRPAZU093 | IMP-1 | - | MBL | >64 | >256 | >256 | >256 | >32 | 48 |
| CRPAZU094 | - | GES-13 | ESBL | >64 | 12 | 48 | 12 | 6 | 32 |
| CRPAZU095 | - | GES-13 | ESBL | >64 | 16 | 32 | 12 | 6 | 16 |
| CRPAZU096 | NDM-1/IMP-1 | - | MBL | >64 | >256 | >256 | >256 | >32 | 16 |
| CRPAZU097 | IMP-1 | - | MBL | >64 | >256 | >256 | >256 | >32 | 24 |
| CRPAZU098 | NDM-1/IMP-1 | - | MBL | >64 | >256 | >256 | >256 | >32 | 24 |
| CRPAZU099 | IMP-1 | - | MBL | >64 | >256 | >256 | >256 | >32 | 64 |
| CRPAZU100 | NDM-1 |  | MBL | >64 | >256 | >256 | >256 | >32 | 4 |
| CRPAZU 101 | - | - | - | >64 | 2 | >256 | >256 | 2 | 64 |
| CRPAZU 102 | VIM-4 | - | MBL | >64 | >256 | >256 | >256 | >32 | 48 |
| CRPAZU 103 | - | - | - | >64 | 6 | 4 | 2 | 1,5 | 3 |
| CRPAZU 104 | - | - | - | >64 | 3 | 48 | 6 | 3 | 16 |
| CRPAZU 105 | VIM-6 | - | MBL | >64 | >256 | >256 | >256 | >32 | >256 |
| CRPAZU 106 | - | PER-1 | ESBL | >64 | 32 | 24 | 4 | 2 | 16 |
| CRPAZU 107 | - | - | - | >64 | 4 | 24 | 8 | 3 | 32 |
| CRPAZU 108 | - | PER-1 | ESBL | >64 | >256 | 16 | 8 | 3 | 24 |
| CRPAZU 109 | - | - | - | >64 | 4 | 12 | 0,75 | 2 | 4 |
| CRPAZU 110 | KPC-2 | - | KPC | >64 | 4 | >256 | 2 | 4 | 4 |
| CRPAZU 111 | - | VEB-1 | ESBL | >64 | >256 | 16 | >256 | 3 | 96 |
| CRPAZU 112 | VIM-11 | - | MBL | >64 | >256 | 4 | >256 | 3 | 16 |
| CRPAZU 113 | - | - | - | >64 | >256 | 32 | >256 | 4 | >256 |
| CRPAZU 114 | VIM-2 | - | MBL | >64 | >256 | >256 | >256 | >32 | 16 |
| CRPAZU 115 | VIM-2 | - | MBL | >64 | >256 | 8 | 96 | >32 | 8 |
| CRPAZU 116 | VIM-2 | - | MBL | >64 | >256 | 32 | >256 | >32 | 6 |
| CRPAZU 117 | VIM-2 | - | MBL | >64 | >256 | >256 | 16 | >32 | 3 |
| CRPAZU 118 | VIM-2 | - | MBL | >64 | >256 | >256 | 32 | >32 | 16 |
| CRPAZU 119 | GES-5 | - | GES-carba | >64 | 16 | >256 | 4 | >32 | 24 |
| CRPAZU 120 | VIM-1 | - | MBL | >64 | >256 | >256 | >256 | >32 | 4 |
| CRPAZU 121 | NDM-1/GES-5 | - | MBL+ GES-carba | >64 | >256 | >256 | >256 | >32 | 24 |
| CRPAZU 122 | NDM-1 | - | MBL | >64 | >256 | >256 | >256 | >32 | 2 |
| CRPAZU 123 | NDM-1 | - | MBL | >64 | >256 | >256 | >256 | >32 | 3 |
| CRPAZU 124 | IMP-15 | - | MBL | >64 | >256 | 6 | >256 | 4 | 6 |
| CRPAZU 125 | SPM-1 | - | MBL | >64 | >256 | >256 | >256 | >32 | 8 |
| CRPAZU 126 | - | - | - | >64 | 3 | 96 | 8 | 1 | 6 |
| CRPAZU 127 | - | - | - | >64 | 4 | 64 | 32 | 4 | >256 |
| CRPAZU 128 | - | - | - | >64 | 2 | 16 | 6 | 3 | 24 |
| CRPAZU 129 | - | - | - | >64 | 3 | 96 | 16 | 4 | 64 |
| CRPAZU 130 | - | - | - | >64 | 3 | 64 | 16 | 3 | 64 |
| CRPAZU 131 | - | - | - | >64 | 2 | 32 | 12 | 3 | 48 |
| CRPAZU 132 | - | - | - | 48 | 3 | 4 | 2 | 0,75 | 8 |
| CRPAZU 133 | - | - | - | >64 | 1,5 | 8 | 4 | 1,5 | 6 |
| CRPAZU 134 | - | - | - | 12 | 2 | 6 | 1,5 | 0,75 | 3 |
| CRPAZU 135 | - | - | - | >64 | 4 | 32 | 32 | 2 | 64 |
| CRPAZU 136 | - | - | - | >64 | 3 | 48 | 16 | 3 | >256 |

C-T, Ceftolozane-Tazobactam; C-A, Ceftazidime-Avibactam, I-R, Imipenem-Relebactam; C-E, Cefepime-Enmetazobactam; M-V, Meropenem-Vaborbactam; A-A, Aztreonam-Avibactam

**Table S5. DD-based susceptibility rates of the CRPA isolates towards first-line antibiotics**

| **Antibiotic** | **EUCAST Breakpoints (mm)** | | **Susceptible (%)** | **Susceptible at increased exposure (%)** | **Resistant (%)** |
| --- | --- | --- | --- | --- | --- |
|  | **S ≥** | **R <** |  |  |  |
| **Piperacillin-tazobactam** | 50 | 18 | - | 3 (2.2%) | 133 (97.8%) |
| **Ceftazidime** | 50 | 17 | - | 0 | 136 (100%) |
| **Cefepime** | 50 | 21 | - | 0 | 136 (100%) |
| **Imipenem** | 50 | 20 | - | 1 (0.7%) | 135 (99.3%) |
| **Meropenem** | 20 | 14 | 4 (3.4%) | 16 (11.8%) | 116 (85.3%) |
| **Aztreonam** | 50 | 18 | - | 45 (33.1%) | 91 (66.9%) |
| **Cefepime*-enmetazobactam** | 50 | 21 | - | 12 (8.8%) | 124 (91.2%) |
| **Ciprofloxacin** | 50 | 26 | - | 25 (18.4%) | 111 (81.6%) |
| **Levofloxacin** | 50 | 18 | - | 19 (14%) | 117 (86%) |
| **Tobramycin** | 18 | 18 | 38 (27.9%) | - | 98 (72%) |
| **Amikacin** | 15 | 15 | 51 (37.5%) | - | 85 (62.5%) |

*For Cefepime-enmetazobactam the CBP for cefepime was considered.

**Table S6.** **MIC-based susceptibility rates of the CRPA isolates towards second-line BL-BLI antibiotics**

| **Antibiotic** | **MIC range (μg/ml)** | **MIC_50_ (μg/ml)** | **MIC_90_ (μg/ml)** | **EUCAST breakpoints** | | **Susceptible (%)** | **Susceptible at increased exposure (%)** | **Resistant (%)** |
| --- | --- | --- | --- | --- | --- | --- | --- | --- |
|  |  |  |  | **S ≤** | **R >** |  |  |  |
| **Ceftazidime-avibactam** | 1 - >256 | >256 | >256 | 8 | 8 | 33 (24.1%) | - | 104 (75.9%) |
| **Ceftolozane-tazobactam** | 1 - >256 | >256 | >256 | 4 | 4 | 30 (21.9%) | - | 107 (78.1%) |
| **Imipenem-relebactam** | 0.5 - >32 | 16 | >32 | 2 | 2 | 29 (21.2%) | - | 108 (78.8%) |
| **Meropenem-relebactam** | 0.5 - >256 | 64 | >256 | 8 | 8 | 16 (11.7%) | - | 121 (88.3%) |
| **Aztreonam-avibactam*** | 0.5 - >256 | 24 | >256 | 0.001 | 16 | - | 66 (48.5%) | 70 (51.5%) |
| **Cefepime-enmetazobactam**** | 12 | >64 | >64 | 0.001 | 8 | - | - | 136 (100%) |

*For aztreonam-avibactam the CBP for aztreonam was considered.

**For Cefepime-enmetazobactam the CBP for cefepime was considered.
